# Fecal Microbiota Transplantation and Health Outcomes: An Umbrella Review of Meta-Analyses of Randomized Controlled Trials

**DOI:** 10.3389/fcimb.2022.899845

**Published:** 2022-06-27

**Authors:** Yapeng Li, Tingting Zhang, Jiahui Sun, Nanyang Liu

**Affiliations:** ^1^ Rehabilitation Therapy Center, Luoyang Orthopedic Hospital of Henan Province, Orthopedic Hospital of Henan Province, Luoyang, China; ^2^ College of First Clinical Medicine, Shandong University of Traditional Chinese Medicine, Jinan, China; ^3^ Graduate School, Beijing University of Chinese Medicine, Beijing, China; ^4^ Xiyuan Hospital, China Academy of Chinese Medical Sciences, Beijing, China

**Keywords:** fecal microbiota transplantation, gut microbiota, umbrella review, meta-analysis, randomized controlled trial

## Abstract

**Background:**

Meta-analysis of randomized clinical trials (RCT) demonstrated several health benefits of fecal microbiota transplantation (FMT). However, there has been little comprehensive assessment of the strength and quality of evidence. We conducted an umbrella review to summarize the evidence of the association between FMT and health outcomes.

**Methods:**

PubMed, Embase, and Cochrane library databases were searched from inception to August 6, 2021. The random-effects model was applied to recalculate the effect estimates. We used AMSTAR 2 and GRADE to assess the methodological quality and to grade the evidence.

**Results:**

A total of 7 meta-analyses comprising 26 RCTs (median [IQR] primary study, 6 [2-7]; median [IQR] sample size, 267 [147-431] participants) were included in the current umbrella review describing 45 unique associations. There were 22 statistically significant associations (49%) demonstrating beneficial outcomes of FMT for antibiotic resistance burden, functional constipation, inflammatory bowel disease, and C. difficile infection. FMT does not appear to be associated with positive outcomes in irritable bowel syndrome and metabolic syndrome. Eight significant associations (36%) were supported by moderate-quality evidence, nine associations (41%) were supported by low-quality evidence, and the remaining associations found to be significant were supported by very low-quality evidence.

**Conclusion:**

Although we found that FMT was positively associated with several outcomes, caution should be exercised in choosing this approach, given the insufficient number of primary studies, low methodological quality, and low quality of evidence. Further high-quality randomized controlled trials with long-term follow-up are needed to improve the strength and credibility of the evidence base.

## Introduction

Accumulating evidence emphasizes the potential contribution of commensal gut microbiota in human health and various gastrointestinal diseases like inflammatory bowel disease (IBD) (Vich et al., 2018; Lavelle and Sokol, 2020), irritable bowel syndrome (IBS) (Pittayanon et al., 2019; Simpson et al., 2020), and gastrointestinal cancer (Kong and Cai, 2019; Lau et al., 2021). It is also well described in non-gastrointestinal diseases, such as cardiovascular (Witkowski et al., 2020), metabolic (Fan and Pedersen, 2021), neurological (Bostanciklioğlu, 2019), and psychiatric diseases (Cheng et al., 2020). In the past two decades, microbiology has developed at an alarming rate, revealing various ways in which these tiny organisms affect our health. Advances in sequencing technology coupled with updates to the microbiome information pipeline have made microbiome analysis cheaper and more complex. In this context, the interaction mechanism between commensal microbiota and these diseases has been revealed gradually. Recent evidence supports the use of antibiotics, prebiotics, probiotics, or fecal microbiota transplantation (FMT) to treat microbiota-associated diseases (Kaźmierczak-Siedlecka et al., 2020), and achieved some impressive results.

**Figure 1:**
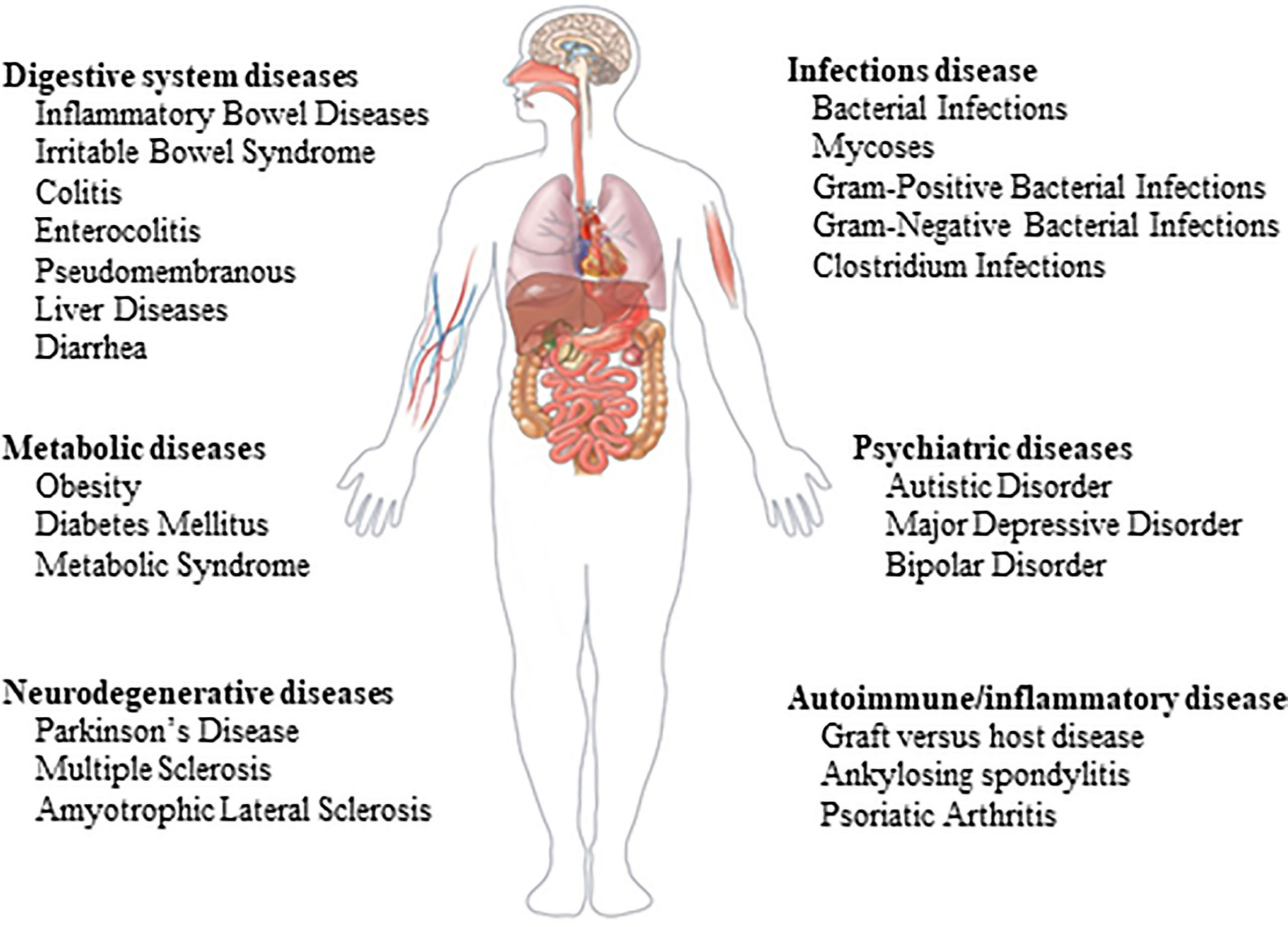
Ongoing clinical trials on fecal microbial transplantation. The data comes from www.clinicaltrial.gov.

FMT is an emerging therapeutic method that has become a research hotspot in biomedicine and clinical medicine (Leshem et al., 2019). The process includes transplanting functional microbiota from healthy individuals into the intestinal tract with pathological microbiota to improve dysbiosis, which thus plays a fundamental role in the treatment of intestinal and extra-intestinal diseases. FMT was originally used to treat pseudomembranous colitis caused by C. difficile infection (CDI). Recently, it has been approved as the standard treatment therapy for recurrent CDI by official guidelines due to its remarkable curative effect (Surawicz et al., 2013). Emerging evidence links gut microbiota disorders with the pathology of numerous diseases (Kelly et al., 2021), prompting researchers to continue to expand the scope of this strategy. According to the latest data from clinicaltrials.gov, nearly 400 trials involving nearly 100 diseases or conditions have been completed or are in progress, most of which were conducted in the past five years **(** **Figure 1** **)**.

Evidence from randomized clinical trials (RCTs) on the efficacy and acceptability of FMT has been obtained *via* both meta-analyses and network meta-analyses (Singh et al., 2021; Cheng et al., 2021; Ramai et al., 2021; Zhou et al., 2021). However, no research has attempted to quantify the credibility of these findings to date. The umbrella review aims to summarize evidence from multiple meta-analyses on the same topic and evaluate sample size, the strength of association, and risk of bias to rank the evidence (Kim J.H. et al., 2020; Barbui et al., 2020; Zhu J. et al., 2020). In this context, we conducted an umbrella review of existing meta-analyses to quantify the strength of the association between FMT and health outcomes. We assessed the methodological quality and potential biases to determine which outcomes are supported by reliable evidence.

## Methods

### Search Strategy

The systematic literature search was conducted in PubMed, Embase, and Cochrane Library from database inception to August 6, 2021, to identify meta-analyses of RCTs investigating the effect of FMT. The search strategy used a combination of the following terms: fecal microbiota transplantation (e.g., intestinal microbiota transfer, fecal transplantation, donor feces infusion) and meta-analysis (e.g., systematic review, meta-analysis, review). No restrictions or filters were applied for the search process. We also manually searched the cited references of the retrieved articles and reviews. Two authors (LYP and ZTT) independently conducted the literature search. Any disagreements were resolved by consultation with a third author (LNY). The detailed search strategy is provided in **Supplementary Table 1**.

### Selection Criteria

Systematic reviews with meta-analyses of RCTs were included. For multiple meta-analyses of the same result, we selected only one meta-analysis for each result to avoid including duplicate studies (Neuenschwander et al., 2019). In this case, we included the largest number of primary studies. If more than one published meta-analysis included the same number of studies, then the one with the largest number of patients was selected. If more than one published meta-analysis meets these two criteria, we selected the one with more available information (e.g., dose-response meta-analysis) (Neuenschwander et al., 2019). When qualified studies contained multiple types of results, we only extracted the pooled effect estimates of RCTs (Hailes et al., 2019; Kim et al., 2019).

Studies were excluded if they were network meta-analyses, if they were systematic reviews without meta-analyses, if the full text of the meta-analysis was not available, or if the meta-analysis lacked data for summary estimates.

### Data Extraction

Two authors (ZTT and SJH) independently extracted data, and disagreements were resolved by consensus. From each meta-analysis, we extracted the first author, journal name, publication year, study design, type of comparison, interesting outcomes, and the number of included studies. We also extracted relative risk (RR) estimates, odds ratio (OR), 95% confidence intervals (CI) and corresponding *P* values, the number of participants and events, follow-up time, meta-analysis models used (fixed effects or random effects), and information on heterogeneity, small-study effects, funding, and conflict of interest. We also extracted any recorded subgroup analysis estimates.

### Assessment of Methodological Quality

We used AMSTAR 2 (A Measurement Tool to Assess Systematic Reviews 2), a strict, validated, and reliable measurement tool, to assess the methodological quality of each meta-analysis (Shea et al., 2017). It consists of 16 items, of which 7 are key items, including quality ratings for meta-analysis of search, reporting, analysis, and transparency (Demurtas et al., 2020). According to the weakness of the key items, the methodological quality was assessed on 4 grades: high, moderate, low, or critically low (Demurtas et al., 2020) **(** **Supplementary Table 2** **)**.

### Evaluation of Quality of Evidence

We used the GRADE (Grading of Recommendations, Assessment, Development, and Evaluation) assessment to evaluate the credibility of the evidence provided by each association in the meta-analysis (Guyatt et al., 2008; Demurtas et al., 2021). Evidence from the meta-analysis of randomized controlled trials was evaluated based on the significance of the pooled effect, using a p-value of <0.05 as statistical significance. The unreported *P-value* was calculated from the 95% confidence interval of the collective effect estimate by using standard methods.

### Statistical Analysis

The effect sizes of individual studies included in each meta-analysis were extracted when the reported data were sufficiently detailed. We used the DerSimonian and Laird random-effects models to recalculate the pooled effect sizes using STATA V.14. (Zhu J. et al., 2020). We did not review the primary study included in each meta-analysis. Heterogeneity between studies was assessed using *I* ^2^ statistics. Values < 50% indicate acceptable heterogeneity, values > 50% suggest moderate heterogeneity, and values > 75% are indicative of high heterogeneity (Zhu J. et al., 2020). Egger’s regression asymmetry test was used to calculate an estimate of publication bias for any reanalysis that included at least 10 studies, which was considered indicative of small-study effects (Demurtas et al., 2020). A p-value < 0.1 was considered statistically significant by Egger’s test.

## Results

### Search Results

**Figure 2:**
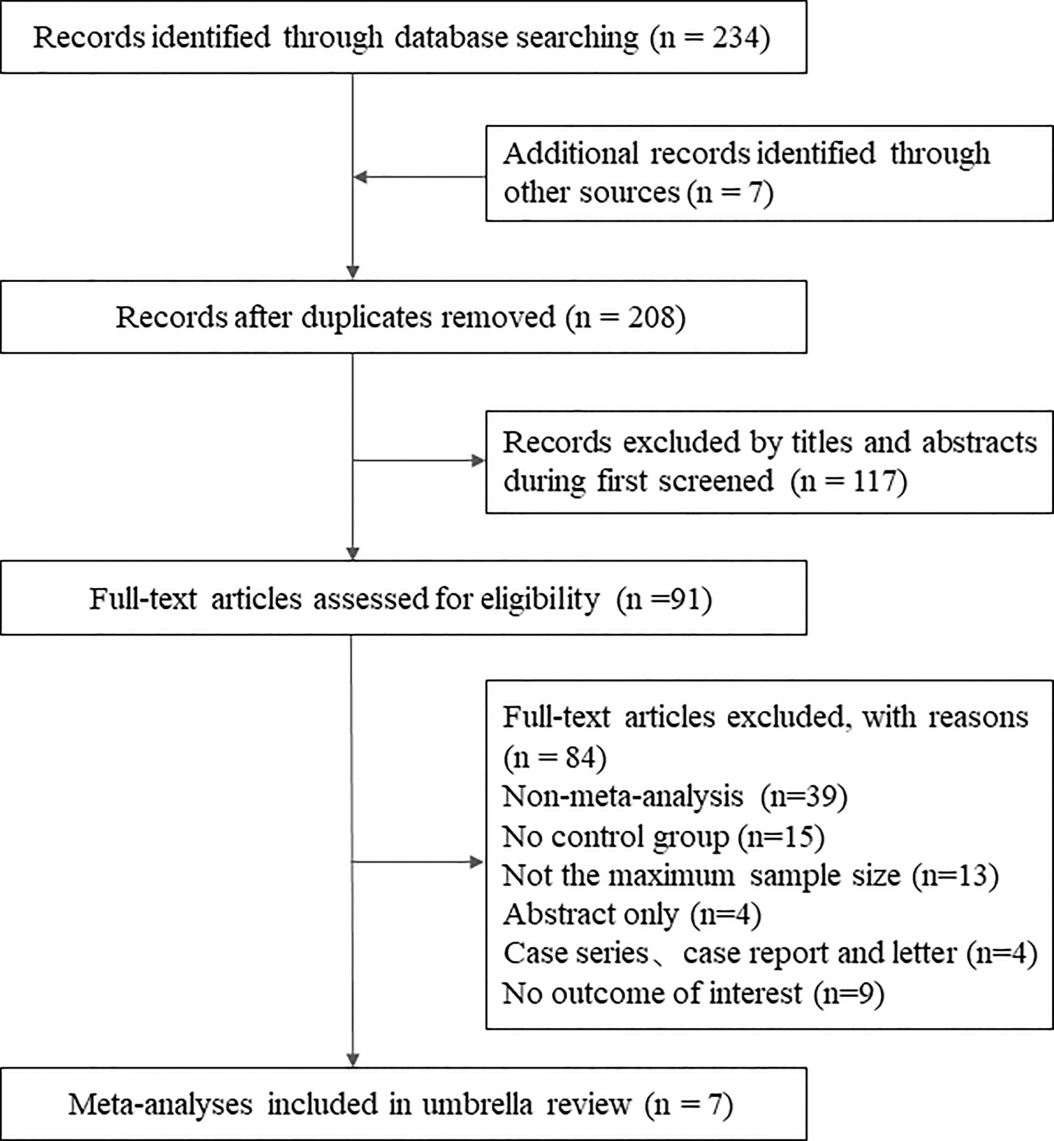
The flowchart of the literature search.

The initial systematic search identified 244 records. After deleting duplicates, we reviewed the titles and abstracts of all retrieved articles, and finally, 91 were determined. Considering the purpose of the present umbrella review, we selected those studies that included the largest number of RCTs. Ultimately, 7 meta-analyses met the eligibility criteria (Hui et al., 2019; Ianiro et al., 2019; Caldeira et al., 2020; Proença et al., 2020; Tang et al., 2020; Dharmaratne et al., 2021; Fang et al., 2021). **Figure 2** shows the flowchart of the literature search. A list of excluded studies can be found in **Supplementary Table 3**.

### Characteristics of Meta-Analyses

The populations considered were in 7 meta-analyses people with CDI (Hui et al., 2019), IBD (Caldeira et al., 2020), IBS (Ianiro et al., 2019), ulcerative colitis (UC) (Tang et al., 2020), functional constipation (Fang et al., 2021), metabolic syndrome (Proença et al., 2020), and antibiotic-resistant burden (Dharmaratne et al., 2021). The median number of primary studies was 6 (interquartile range, 2-7), and the median number of cases was 267 (interquartile range, 147-431) **(** **Table 1** **)**.

**Table 1:** The general characteristics of the included meta-analysis.

Source	Disease	Year	Intervention group	Control group	No. of primary studies	No.of cases	Follow-up time (wks)	Outcome
Dharmaratne	Antibiotic resistance burden	2021	FMT	placebo	2	59	28	Clinical remission
Fang	Functional constipation	2021	FMT+ laxative	laxative	2	163	4-12	Total effective rate, BFSF score, Wexner score, KESS score, PAC-QOL score, adverse effects
de Fàtima Caldeira	Inflammatory bowel disease	2019	FMT	placebo	6	355	7-12	Clinical remission, clinical response, adverse event
Tang	Ulcerative colitis	2020	FMT	placebo	7	431	4-48	Clinical remission rate, adverse events, multi-donor, single-donor, lower digestive tract, up digestive tract, frozen feces, fresh feces
Hui	Clostridium difficile infection	2019	FMT	placebo or vancomycin	8	537	8-24	Clinical remission, frequency of infusion (multiple infusions and single infusion), adverse events
Ianiro	Irritable bowel syndrome	2019	FMT	placebo	5	267	8-48	Clinical remission, adverse events, adverse events (FMT *via* capsules), adverse events (FMT *via* colonoscopy), FMT *via* oral capsules, FMT *via* colonoscopy, FMT *via* nasojejunal tube
Proença	Metabolic Syndrome	2020	FMT	placebo	6	147	2-12	HbA1c, HDL cholesterol, LDL cholesterol, fasting glucose, triglycerides, total cholesterol, BMI, weight, HOMA-IR, adverse events, hip width

Abbreviations: BFSF, bristol stool form scale; BMI, body mass index; FMT, fecal microbiota transplantation; HbA1c, Hemoglobin A1c; HDL, high density lipoprotein; HOMA-IR, homeostatic model assessment of insulin resistance; KESS, knowles eccersley scott symptom; LDL, low density lipoprotein; MD, mean difference; NA, not applicable; PAC-QOL, patient assessment of constipation quality of life uestionnaire; RR, risk ratio; 95%CI, 95% confidence intervals.

### Methodological Quality

Results of AMSTAR 2 for each meta-analysis were presented in **Table 2**. Overall, the methodological quality assessment of 7 studies was determined to be critically low [6 studies (85.7%)] or low [1 studies (14.3%)]. The most common critical flaws were the absence of a detailed literature exclusion list and funding sources and did not consider the risk of bias and heterogeneity when preparing conclusions and recommendations. However, publication bias was not evaluated due to the insufficient number of primary studies (less than 10), which may magnify the low methodological quality.

**Table 2:** The methodological quality of included meta-analysis using AMSTAR-2.

Source	Item1	Item2	Item3	Item4	Item5	Item6	Item7	Item8	Item9	Item10	Item11	Item12	Item13	Item14	Item15	Item16	Overall quality
Tang	Y	Y	Y	PY	Y	Y	N	Y	Y	N	Y	Y	Y	N	N	Y	Critically low
Dharmaratne	Y	Y	Y	PY	Y	Y	N	Y	N	N	Y	N	N	Y	N	Y	Critically low
Ianiro	Y	Y	Y	Y	Y	Y	N	Y	Y	N	Y	N	N	Y	N	Y	Critically low
Proença	Y	Y	Y	PY	N	Y	N	Y	Y	N	Y	N	N	Y	N	Y	Critically low
Hui	Y	Y	Y	PY	N	Y	N	Y	Y	N	Y	Y	Y	Y	Y	Y	Low
Fang	Y	Y	Y	PY	Y	Y	N	Y	Y	N	Y	N	N	N	N	Y	Critically low
de Fàtima Caldeira	Y	Y	Y	PY	Y	Y	PY	Y	Y	N	Y	N	N	Y	N	Y	Critically low

Rationale for selection of items:1. Did the research questions and inclusion criteria for the review include the components of PICO?2. Did the report of the review contain an explicit statement that the review methods were established prior to the conduct of the review and did the report justify any significantdeviations from the protocol?3. Did the review authors explain their selection of the study designs for inclusion in the review?4. Did the review authors use a comprehensive literature search strategy?5. Did the review authors perform study selection in duplicate?6. Did the review authors perform data extraction in duplicate?7. Did the review authors provide a list of excluded studies and justify the exclusions?8. Did the review authors describe the included studies in adequate detail?9. Did the review authors use a satisfactory technique for assessing the risk of bias (RoB) in individual studies that were included in the review?10. Did the review authors report on the sources of funding for the studies included in the review?11. If meta-analysis was performed, did the review authors use appropriate methods for statistical combination of results?12. If meta-analysis was performed, did the review authors assess the potential impact of RoB in individual studies on the results of the meta-analysis or other evidence synthesis?13. Did the review authors account for RoB in primary studies when interpreting/discussing the results of the review?14. Did the review authors provide a satisfactory explanation for, and discussion of, any heterogeneity observed in the results of the review?15. If they performed quantitative synthesis did the review authors carry out an adequate investigation of publication bias (small study bias) and discuss its likely impact on the results of the review?16. Did the review authors report any potential sources of conflict of interest, including any funding they received for conducting the review?Y, yes; N: no; PY, partial yes

### Description and Summary of Associations

Associations analyzed included 7 clinical effect outcomes (ie, clinical remission, clinical response, and total effective rate), 8 adverse events, 8 lipid profile outcomes (ie, HDL cholesterol, LDL cholesterol, total cholesterol, and triglycerides), 3 glycemic profile outcomes [ie, fasting plasma glucose, hemoglobin A1c, and homeostatic model assessment of insulin resistance (HOMA-IR)], 5 anthropometric measures [ie, hip-width, weight, and body mass index (BMI)], 4 stool measures [ie, bristol stool form scale (BSFS), Wexner score, Knowles Eckersley Scott symptom (KESS), patient assessment of constipation quality of life (PAC-QOL)], 3 FMT formulation outcomes (ie, oral capsules, frozen feces, and fresh feces), 4 FMT method outcomes (ie, colonoscopy, nasojejunal tube, lower digestive tract, and upper digestive tract), and 3 FMT donor outcomes (multi-donor and single-donor) **(** **Table 1** **)**.

Strength of evidence of the 45 associations assessed using GRADE found that 23 associations (51%) were supported by low evidence strength, while the remaining associations were supported by moderate [11 associations (24%)] and very low [7 associations (16%)], respectively. Four of these associations cannot be assessed (9%) (**Supplementary Table 4**). Recalculation using random-effect model yielded 22 (49%) statistically significant associations (*p* < 0.05). Nine (49%) of which were supported by low evidence, followed by moderate [8 associations (36%)] and very low evidence [5 associations (23%)] (**Table 3**). These associations indicated beneficial outcomes associated with FMT for antibiotic resistance burden, functional constipation, IBD, UC, and CDI, while metabolic syndrome and IBS did not.

**Table 3:** Summary of significant associations of fecal microbiota transplantation with health outcomes.

Source	Disease	Outcome	No. of primary studies	No.of cases	Duration of treatment (wks)	Metric	Random effect estimates(Intervention group VS Control group)	*p*-value	*I* ^2^ (%)	GRADE rating	AMSTAR-2 rating
Dharmaratne	Antibiotic resistance burden	Clinical remission	2	59	28	RR	RR = 4.90; 95% CI (1.92-12.50)	0.0003	0	Low	Critically low
Fang	Functional constipation	Total effective rate	2	163	4-12	RR	RR=1.33, 95% CI (1.10, 1.59)	0.003	13	Low	Critically low
		BFSF score	3	206	4-12	MD	MD=1.04, 95% CI (0.57, 1.51)	<0.001	76	Very low	
		Wexner score	2	146	4-12	MD	MD=-3.25, 95% CI (-5.58, -0.92)	0.006	92	Very low	
		KESS score	2	160	4-12	MD	MD=-5.75, 95% CI (-7.64, -3.68)	<0.001	0	Very low	
		PAC-QOL score	3	246	4-12	MD	MD=-18.56, 95% CI (-24.63, -10.68)	<0.001	78	Very low	
de Fàtima Caldeira	Inflammatory bowel disease	Clinical remission	6	355	7-12	RR	RR=1.70, 95% CI (1.12, 2.56)	0.029	45	Moderate	Critically low
		Clinical response	6	355	7-12	RR	RR=1.68, 95% CI (1.04, 2.72)	0.042	55	Moderate	
Tang	Ulcerative colitis	Clinical remission	7	431	4-48	RR	RR =1.50, 95% CI (1.06–2.12)	0.02	48	Moderate	Critically low
		Multi-donor	4	267	4-48	RR	RR = 2.07, 95% CI (1.16–3.71)	0.01	41	Low	
		Single-donor	2	76	4-48	RR	RR = 1.30, 95% CI (0.90–1.73)	0.07	0	Low	
		Lower digestive tract	5	368	4-48	RR	RR = 1.68, 95% CI (1.09–2.59)	0.02	65	Low	
		Frozen feces	4	263	4-48	RR	RR = 1.60, 95% CI (1.02-2.59)	0.04	59	Low	
Hui	Clostridium difficile infection	Clinical remission	8	537	8-24	RR	RR =1.82, 95% CI, 1.19–2.78	0.002	76	Moderate	Low
		Donor (multiple vs single)	4	326	8-24	RR	RR =1.21, 95% CI, 1.08–1.37	0.001	0	Low	
Ianiro	Irritable bowel syndrome	FMT *via* oral capsules	2	100	8-48	RR	RR=1.96, 95% CI (1.19‐3.20)	0.008	14	Low	Critically low
		FMT *via* colonoscopy	2	103	8-48	RR	RR=0.63, 95% CI (0.43‐0.93)	0.02	0	Low	
Proença	Metabolic syndrome	HDL cholesterol	6	146	2-6	MD	(MD = 0.09, 95% CI (0.02, 0.16)	0.01	0	Moderate	Critically low
		LDL cholesterol	6	146	2-6	MD	(MD = 0.19, 95% CI (0.05, 0.34)	0.008	0	Moderate	

BFSF, bristol stool form scale; FMT, fecal microbiota transplantation; HDL, high density lipoprotein; KESS, knowles eccersley scott symptom; LDL, low density lipoprotein; MD, ean difference; PAC-QOL, patient assessment of constipation quality of life questionnaire; RR, risk ratio; 95% CI, 95% confidence intervals.

#### Antibiotic Resistance Burden

Two primary studies evaluated the therapeutic effect of FMT on the antibiotic resistance burden, including one unique outcome. Random-effects model results show that FMT has statistical significance for the antibiotic resistance burden (RR=4.90, 95% CI=1.92 to 12.50), which is considered low evidence.

#### Functional Constipation

Six associations of functional constipation were evaluated, of which four were supported by very low evidence, followed by one low evidence. The strength of evidence for safety cannot be assessed. Five associations are statistically significant, indicating the positive benefits of FMT on functional constipation.

#### Inflammatory Bowel Disease

Six primary studies documented the impact of FMT on IBD involved 3 associations. Two associations had moderate-quality evidence, in this meta-analysis that found FMT was associated with increased clinical remission (RR=1.70, 95% CI=1.12 to 2.56) and clinical response (RR=1.68, 95% CI=1.04 to 2.72) in patients with IBD compared with placebo. The strength of evidence for safety cannot be assessed.

#### Ulcerative Colitis

Current literature also reports the contribution of FMT to UC. Six statistically significant associations were supported by low to moderate quality of evidence. FMT was associated with increased clinical remission (RR=1.50, 95% CI=1.06 to 2.12) in patients with IBD compared with placebo. Single donor (RR=1.30, 95% CI =0.90 to 1.73) and multiple donors (RR=2.07, 95% CI=1.16 to 3.71) share the same validity. However, transplantation *via* lower digestive tract (RR=1.68, 95% CI=1.09 to 2.59) appears to be more effective than up digestive tract (RR=0.99, 95% CI=0.47 to 2.09), and frozen feces (RR=1.60, 95% CI=1.02 to 2.59) are more effective than fresh feces (RR=2.38, 95% CI=0.62 to 9.11). There was no statistical difference in adverse events (RR=1.21, 95% CI=0.86 to 1.70) between FMT and placebo, supported by moderate-quality evidence.

#### C. Difficile Infection

Treatment of CDI with FMT was evaluated in 8 primary studies involving 2 associations. This meta-analysis found that FMT was associated with increased clinical remission (RR=1.82, 95% CI=1.19 to 2.78) in patients with IBD compared with placebo or vancomycin, supported by moderate-quality evidence. However, multiple infusions are more effective than single infusions (RR=1.21, 95% CI=1.08 to 1.37).

#### Irritable Bowel Syndrome

Among the 7 associations supported by very low to moderate-quality evidence, only 2 were statistically significant. One association had moderate-quality evidence found that FMT was not associated with clinical remission in patients with IBS compared with placebo (RR=0.98, 95% CI=0.58 to 1.66). Another association with moderate-quality evidence suggested no difference in adverse events between placebo and FMT (RR=0.93, 95% CI=0.45 to 1.92). Two significantly associated outcomes suggested that oral FMT capsules appeared to be more effective (RR=1.96, 95% CI=1.19 to 3.20), while *via* colonoscopy was less effective than placebo (RR=0.63, 95% CI=0.43 to 0.93).

#### Metabolic Syndrome

Metabolic syndrome refers to a pathological state in which proteins, fats, and carbohydrates are disordered, such as obesity and insulin resistance. After 2-6 months follow-up, HDL cholesterol (MD=0.09, 95% CI=0.02 to 0.16) and LDL cholesterol (MD=0.19, 95% CI=0.05 to 0.34) were the only FMT types associated with statistically significant in participants with overweight or obesity compared with placebo, supported by moderate evidence. However, the effect became insignificant after 12 months of follow-up.

### Heterogeneity Between Primary Studies

Of all 45 associations, 24 had acceptable heterogeneity (<50%), 14 had significant heterogeneity (>50%), and another 7 could not be assessed. Associations initially graded as very low to moderate quality retained the same rank when removing RCTs with significant heterogeneity.

### Publication Bias and Small Study-Effects

The publication bias and small study effects for each meta-analysis were evaluated by Egger tests. Unfortunately, all the associations included fewer than 10 primary studies. Therefore, the contribution of small-study effects to the quality of evidence was not assessed.

## Discussion

This study is the first umbrella review that systematically assessed the role of FMT in several health outcomes by incorporating the evidence from the current meta-analysis of RCTs and evaluated the evidence by using well-recognized GRADE criteria. Our findings are valuable in the context of the lack of evidence-based support and standards for FMT strategies that inform clinicians and the general population. We used the random-effects model to recalculate each association for better comparison across outcomes. Moreover, we used standard approaches to assess the methodological quality of meta-analyses and the quality of evidence for each association. Furthermore, we performed sensitivity analyses and small study effects analyses to further strengthen the reliability of the results.

The gut microbiota refers to the bacteria, viruses, parasites, and fungi colonizing the intestinal tract (Larabi et al., 2020). The adult gut microbiota is composed of more than 2000 bacterial species to form a diversified, stable, resistant, and elastic microbial ecosystem that participates in host immunity, metabolism, and other biological functions (Fung et al., 2017; Kim M.S. et al., 2020). Dysbiosis is disturbances in the function and composition of the microbiota driven by environmental and host-related factors (Leshem et al., 2019). This process may be involved in the pathogenesis of many diseases, such as IBD, IBS (Cui et al., 2021), multiple sclerosis (Engen et al., 2020; Li et al., 2020), hepatic encephalopathy (Kao et al., 2016; Madsen et al., 2021), cancer (Chen et al., 2019; Kaźmierczak-Siedlecka et al., 2020; Zhu H. et al., 2020) and metabolic syndrome (Yu et al., 2020). Targeting the disturbed microbiota, which may be achieved by dietary interventions, probiotics, prebiotics, antibiotics, and FMT, might affect the progress of these conditions. Research related to FMT can obtain the most convincing evidence that gut microbiota plays a role in human diseases. (Leshem et al., 2019).

The application of stool therapy can be traced back to ancient Chinese medicine nearly 1700 years ago (Zhang et al., 2018). (Eiseman et al., 1958) first reported FMT as an adjuvant treatment for patients with antibiotic-associated diarrhea, which opened the door to the modern era. Subsequent reports confirmed that C. difficile was the culprit responsible for post-antibiotic colitis (known today as pseudomembranous colitis) (Kelly and Lamont, 2008; Freeman et al., 2010). Following these revelations, numerous trials indicated the clinical effect of FMT on pseudomembranous colitis caused by CDI and finally approved it as a standard treatment strategy by official guidelines (Surawicz et al., 2013). With the continuous advancement of gut microbiota research, the underlying mechanisms of many conditions have been linked. Correcting the imbalanced gut microbiota is also becoming a potential alternative strategy. Recently, FMT treatment attempts have gradually expanded from the initial gastrointestinal disorder to other diseases, such as the nervous system and cardiovascular system. Additionally, the establishment of a stool bank makes FMT an easily available and useful option.

In this umbrella review, we found 45 unique associations. Of these, 22 statistically significant associations (49%) were distributed across assessments of antibiotic resistance burden, functional constipation, IBD, and CDI. In contrast, few significant associations were found in metabolic syndrome and IBS. Evidence for statistically significant associations ranges from very low to moderate. None of the associations were supported by high-quality evidence. FMT was mostly successful in the initial phase (ie, 4-24 weeks). Although participants may frequently experience a plateau afterward, some effects persisted after longer follow-up (ie, 24-48 weeks). For safety, although 8 associations were assessed, 4 did not record specific effect estimates. Of the remaining 4 associations, 3 showed no statistically significant difference in adverse events between FMT and placebo. Furthermore, common adverse events, such as bloating, diarrhea, nausea, abdominal pain, and fever, were mild and resolved on their own.

This umbrella review used the AMSTAR 2 tool to assess the methodological quality of meta-analysis and identified several potential flaws, like inaccurate assessments of risk of bias and heterogeneity, a lack of funding sources, and literature exclusion lists. These flaws lead to the low quality of the evidence from primary studies, thereby affecting the overall quality (low or critically low) of the meta-analysis. Insufficient information on randomization, allocation concealment and blinding are the main factors that downgrade the quality of evidence. This was followed by small sample size and significant heterogeneity. We did not find any convincing factors to upgrade the quality of evidence. Of note, future meta-analyses in this field should use AMSTAR 2 as an executive checklist to ensure high-quality evidence. Additionally, the risk of bias for most of the associations was rated as very severe according to GRADE criteria. These associations were ultimately rated at high risk of bias in part because of incomplete blinding, which may have affected treatment assignments and outcome measures between groups. The limited number of primary studies and participants was also a major contributor to the high risk of bias.

Our review yielded several key messages of high interest to clinicians and patients, especially those contemplating FMT strategies. It is important to emphasize that despite multiple preclinical evidence supporting the health benefits of FMT, evidence of clear and sustainable clinical benefits is still lacking. Furthermore, although this novel approach seems safe and easy to implement, we should be cautious because the long-term effects are still unknown or unrecognized. Moreover, as an emerging medical therapeutic strategy, FMT is not yet a standardized treatment method. The protocols vary according to local procedures. Uniform standards not established on fecal formulation, transplantation method and frequency may be the reason for the inconsistent results. Therefore, well-designed studies are strongly needed to investigate the long-term efficacy and safety outcomes of FMT.

## Limitations

Potential limitations should be considered when interpreting the results of our work. First, we used pre-established tools to assess the quality of meta-analysis, which relies on complete data in the primary study. Although the two authors conducted the assessment back-to-back, subjectivity was inevitable. Second, we did not include meta-analyses of observational studies that may have had a longer follow-up. Most of the RCTs included in our analysis were limited to short-term follow-up and relatively small sample sizes. Therefore, follow-up assessments of continued beneficial effects after cessation of FMT are lacking. Third, Insufficient number of primary studies and methodological flaws may limit the true understanding of FMT. Fourth, we used DerSimonian and Laird’s random-effects method to calculate the aggregate hazard ratio and the corresponding 95% CI to ensure comparability with the previous meta-analysis. However, future meta-analyses should use the Hartung-Knapp method, which can better reflect the uncertainty of the differences between studies, expressed with a wider confidence interval. Fifth, existing meta-analyses documented fewer adverse events, which prevented us from systematically assessing the safety of FMT.

## Conclusion

This umbrella review found beneficial associations of FMT with several health outcomes. Although our work highlights the significance of the use of FMT by public health authorities in some diseases, the quality of evidence is less convincing due to insufficient numbers of primary studies and low methodological quality. Continued research into the therapeutic effects of FMT is important. In addition to large-scale RCTs, well-designed long-term follow-up protocols must also be considered to evaluate longer-term efficacy and safety.

## Author Contributions

NL and YL conducted the literature search, study selection and data extraction and analysis. TZ and JS contributed to the data interpretation, writing, and editing of the manuscript. All authors have read and agreed to the published version of the manuscript.

## Conflict of Interest

The authors declare that the research was conducted in the absence of any commercial or financial relationships that could be construed as a potential conflict of interest. 

## Publisher’s Note

All claims expressed in this article are solely those of the authors and do not necessarily represent those of their affiliated organizations, or those of the publisher, the editors and the reviewers. Any product that may be evaluated in this article, or claim that may be made by its manufacturer, is not guaranteed or endorsed by the publisher.

## References

[B1] Barbui C. Purgato M. Abdulmalik J. Acarturk C. Eaton J. Gastaldon C.. (2020). Efficacy of Psychosocial Interventions for Mental Health Outcomes in Low-Income and Middle-Income Countries: An Umbrella Review. Lancet Psychiatry 7 (2), 162–172. doi: 10.1016/S2215-0366(19)30511-5 31948935

[B2] Bostanciklioğlu M. (2019). The Role of Gut Microbiota in Pathogenesis of Alzheimer's Disease. J. Appl. Microbiol. 127 (4), 954–967 doi: 10.1111/jam.14264 30920075

[B3] Caldeira D. F. Borba H. H. Tonin F. S. Wiens A. Fernandez-Llimos F. Pontarolo R. (2020). Fecal Microbiota Transplantation in Inflammatory Bowel Disease Patients: A Systematic Review and Meta-Analysis. PLos One 15 (9), e0238910. doi: 10.1371/journal.pone.0238910 32946509 PMC7500646

[B4] Cheng S. Han B. Ding M. Wen Y. Ma M. Zhang L.. (2020). Identifying Psychiatric Disorder-Associated Gut Microbiota Using Microbiota-Related Gene Set Enrichment Analysis. Brief. Bioinform. 21 (3), 1016–1022. doi: 10.1093/bib/bbz034 30953055

[B5] Cheng F. Huang Z. Wei W. Li Z. (2021). Fecal Microbiota Transplantation for Crohn's Disease: A Systematic Review and Meta-Analysis. Tech. Coloproctol. 25 (5), 495–504. doi: 10.1007/s10151-020-02395-3 33759066

[B6] Chen D. Wu J. Jin D. Wang B. Cao H. (2019). Fecal Microbiota Transplantation in Cancer Management: Current Status and Perspectives. Int. J. Cancer 145 (8), 2021–2031. doi: 10.1002/ijc.32003 30458058 PMC6767494

[B7] Cui J. Lin Z. Tian H. Yang B. Zhao D. Ye C.. (2021). Term Follow-Up Results of Fecal Microbiota Transplantation for Irritable Bowel Syndrome: A Single-Center, Retrospective Study. Front. Med. 8. doi: 10.3389/fmed.2021.710452 PMC8362996 34395484

[B8] Demurtas J. Celotto S. Beaudart C. Sanchez-Rodriguez D. Balci C. Soysal P.. (2020). The Efficacy and Safety of Influenza Vaccination in Older People: An Umbrella Review of Evidence From Meta-Analyses of Both Observational and Randomized Controlled Studies. Ageing Res. Rev. 62, 101118. doi: 10.1016/j.arr.2020.101118 32565328

[B9] Demurtas J. Fanelli G. N. Romano S. L. Solari M. Yang L. Soysal P.. (2021). Stem Cells for Treatment of Cardiovascular Diseases: An Umbrella Review of Randomized Controlled Trials. Ageing Res. Rev. 67, 101257. doi: 10.1016/j.arr.2021.101257 33434684

[B10] Dharmaratne P. Rahman N. Leung A. Ip M. (2021). Is There a Role of Faecal Microbiota Transplantation in Reducing Antibiotic Resistance Burden in Gut? A Systematic Review and Meta-Analysis. Ann. Med. (Helsinki) 53 (1), 662–681. doi: 10.1080/07853890.2021.1927170 PMC8238059 34170204

[B11] Eiseman B. Silen W. Bascom G. S. Kauvar A. J. (1958). Fecal Enema as an Adjunct in the Treatment of Pseudomembranous Enterocolitis. Surgery 44 (5), 854–859. 13592638

[B12] Engen P. A. Zaferiou A. Rasmussen H. Naqib A. Green S. J. Fogg L. F.. (2020). Single-Arm, Non-Randomized, Time Series, Single-Subject Study of Fecal Microbiota Transplantation in Multiple Sclerosis. Front. Neurol. 11. doi: 10.3389/fneur.2020.00978 PMC7506051 33013647

[B13] Fang S. Wu S. Ji L. Fan Y. Wang X. Yang K. (2021). The Combined Therapy of Fecal Microbiota Transplantation and Laxatives for Functional Constipation in Adults. Medicine 100 (14), e25390. doi: 10.1097/MD.0000000000025390 33832129 PMC8036125

[B14] Fan Y. Pedersen O. (2021). Gut Microbiota in Human Metabolic Health and Disease. Nat. Rev. Microbiol. 19 (1), 55–71. doi: 10.1038/s41579-020-0433-9 32887946

[B15] Freeman J. Bauer M. P. Baines S. D. Corver J. Fawley W. N. Goorhuis B.. (2010). The Changing Epidemiology of Clostridium Difficile Infections. Clin. Microbiol. Rev. 23 (3), 529–549. doi: 10.1128/CMR.00082-09 20610822 PMC2901659

[B16] Fung T. C. Olson C. A. Hsiao E. Y. (2017). Interactions Between the Microbiota, Immune and Nervous Systems in Health and Disease. Nat. Neurosci. 20 (2), 145–155. doi: 10.1038/nn.4476 28092661 PMC6960010

[B17] Guyatt G. H. Oxman A. D. Vist G. E. Kunz R. Falck-Ytter Y. Alonso-Coello P.. (2008). GRADE: An Emerging Consensus on Rating Quality of Evidence and Strength of Recommendations. BMJ 336 (7650), 924–926. doi: 10.1136/bmj.39489.470347.AD 18436948 PMC2335261

[B18] Hailes H. P. Yu R. Danese A. Fazel S. (2019). Long-Term Outcomes of Childhood Sexual Abuse: An Umbrella Review. Lancet Psychiatry 6 (10), 830–839. doi: 10.1016/S2215-0366(19)30286-X 31519507 PMC7015702

[B19] Hui W. Li T. Liu W. Zhou C. Gao F. (2019). Fecal Microbiota Transplantation for Treatment of Recurrent C. Difficile Infection: An Updated Randomized Controlled Trial Meta-Analysis. PLos One 14 (1), e0210016. doi: 10.1371/journal.pone.0210016 30673716 PMC6343888

[B20] Ianiro G. Eusebi L. H. Black C. J. Gasbarrini A. Cammarota G. Ford A. C. (2019). Systematic Review With Meta-Analysis: Efficacy of Faecal Microbiota Transplantation for the Treatment of Irritable Bowel Syndrome. Aliment. Pharm. Ther. 50 (3), 240–248. doi: 10.1111/apt.15330 31136009

[B21] Kao D. Roach B. Park H. Hotte N. Madsen K. Bain V.. (2016). Fecal Microbiota Transplantation in the Management of Hepatic Encephalopathy. Hepatology 63 (1), 339–340. doi: 10.1002/hep.28121 26264779

[B22] Kaźmierczak-Siedlecka K. Daca A. Fic M. van de Wetering T. Folwarski M. Makarewicz W. (2020). Therapeutic Methods of Gut Microbiota Modification in Colorectal Cancer Management - Fecal Microbiota Transplantation, Prebiotics, Probiotics, and Synbiotics. Gut Microbes 11 (6), 1518–1530. doi: 10.1080/19490976.2020.1764309 32453670 PMC7524363

[B23] Kelly C. P. Lamont J. T. (2008). Clostridium Difficile–More Difficult Than Ever. N Engl. J. Med. 359 (18), 1932–1940. doi: 10.1056/NEJMra0707500 18971494

[B24] Kelly C. R. Yen E. F. Grinspan A. M. Kahn S. A. Atreja A. Lewis J. D.. (2021). Fecal Microbiota Transplantation Is Highly Effective in Real-World Practice: Initial Results From the FMT National Registry. Gastroenterology 160 (1), 183–192.e3. doi: 10.1053/j.gastro.2020.09.038 33011173 PMC8034505

[B25] Kim M. S. Kim Y. Choi H. Kim W. Park S. Lee D.. (2020). Transfer of a Healthy Microbiota Reduces Amyloid and Tau Pathology in an Alzheimer's Disease Animal Model. Gut 69 (2), 283–294. doi: 10.1136/gutjnl-2018-317431 31471351

[B26] Kim J. H. Kim J. Y. Lee J. Jeong G. H. Lee E. Lee S.. (2020). Environmental Risk Factors, Protective Factors, and Peripheral Biomarkers for ADHD: An Umbrella Review. Lancet Psychiatry 7 (11), 955–970. doi: 10.1016/S2215-0366(20)30312-6 33069318

[B27] Kim J. Y. Son M. J. Son C. Y. Radua J. Eisenhut M. Gressier F.. (2019). Environmental Risk Factors and Biomarkers for Autism Spectrum Disorder: An Umbrella Review of the Evidence. Lancet Psychiatry 6 (7), 590–600. doi: 10.1016/S2215-0366(19)30181-6 31230684

[B28] Kong F. Cai Y. (2019). Study Insights Into Gastrointestinal Cancer Through the Gut Microbiota. BioMed. Res. Int. 2019, 8721503. doi: 10.1155/2019/8721503 31341907 PMC6612970

[B29] Larabi A. Barnich N. Nguyen H. (2020). New Insights Into the Interplay Between Autophagy, Gut Microbiota and Inflammatory Responses in IBD. Autophagy 16 (1), 38–51. doi: 10.1080/15548627.2019.1635384 31286804 PMC6984609

[B30] Lau H. Sung J. J. Yu J. (2021). Gut Microbiota: Impacts on Gastrointestinal Cancer Immunotherapy. Gut Microbes 13 (1), 1–21. doi: 10.1080/19490976.2020.1869504 PMC7808428 33435800

[B31] Lavelle A. Sokol H. (2020). Gut Microbiota-Derived Metabolites as Key Actors in Inflammatory Bowel Disease. Nat. Rev. Gastroenterol. Hepatol. 17 (4), 223–237. doi: 10.1038/s41575-019-0258-z 32076145

[B32] Leshem A. Horesh N. Elinav E. (2019). Fecal Microbial Transplantation and Its Potential Application in Cardiometabolic Syndrome. Front. Immunol. 10. doi: 10.3389/fimmu.2019.01341 PMC6587678 31258528

[B33] Li K. Wei S. Hu L. Yin X. Mai Y. Jiang C.. (2020). Protection of Fecal Microbiota Transplantation in a Mouse Model of Multiple Sclerosis. Mediators Inflammation 2020, 2058272. doi: 10.1155/2020/2058272 PMC7426773 32831634

[B34] Madsen M. Kimer N. Bendtsen F. Petersen A. M. (2021). Fecal Microbiota Transplantation in Hepatic Encephalopathy: A Systematic Review. Scand. J. Gastroenterol. 56 (5), 560–569. doi: 10.1080/00365521.2021.1899277 33840331

[B35] Neuenschwander M. Ballon A. Weber K. S. Norat T. Aune D. Schwingshackl L.. (2019). Role of Diet in Type 2 Diabetes Incidence: Umbrella Review of Meta-Analyses of Prospective Observational Studies. BMJ 366, l2368. doi: 10.1136/bmj.l2368 31270064 PMC6607211

[B36] Pittayanon R. Lau J. T. Yuan Y. Leontiadis G. I. Tse F. Surette M.. (2019). Gut Microbiota in Patients With Irritable Bowel Syndrome-A Systematic Review. Gastroenterology 157 (1), 97–108. doi: 10.1053/j.gastro.2019.03.049 30940523

[B37] Proença I. M. Allegretti J. R. Bernardo W. M. de Moura D. T. H. Ponte Neto A. M. Matsubayashi C. O.. (2020). Fecal Microbiota Transplantation Improves Metabolic Syndrome Parameters: Systematic Review With Meta-Analysis Based on Randomized Clinical Trials. Nutr. Res. 83, 1–14. doi: 10.1016/j.nutres.2020.06.018 32987284

[B38] Ramai D. Zakhia K. Fields P. J. Ofosu A. Patel G. Shahnazarian V.. (2021). Fecal Microbiota Transplantation (FMT) With Colonoscopy Is Superior to Enema and Nasogastric Tube While Comparable to Capsule for the Treatment of Recurrent Clostridioides Difficile Infection: A Systematic Review and Meta-Analysis. Dig Dis. Sci. 66 (2), 369–380. doi: 10.1007/s10620-020-06185-7 32166622

[B39] Shea B. J. Reeves B. C. Wells G. Thuku M. Hamel C. Moran J.. (2017). AMSTAR 2: A Critical Appraisal Tool for Systematic Reviews That Include Randomised or non-Randomised Studies of Healthcare Interventions, or Both. BMJ 358, j4008. doi: 10.1136/bmj.j4008 28935701 PMC5833365

[B40] Simpson C. A. Mu A. Haslam N. Schwartz O. S. Simmons J. G. (2020). Feeling Down? A Systematic Review of the Gut Microbiota in Anxiety/Depression and Irritable Bowel Syndrome. J. Affect. Disord.266, 429–446. doi: 10.1016/j.jad.2020.01.124 32056910

[B41] Singh T. Bedi P. Bumrah K. Gandhi D. Arora T. Verma N.. (2021). Fecal Microbiota Transplantation and Medical Therapy for Clostridium Difficile Infection: Meta-Analysis of Randomized Controlled Trials. J. Clin. Gastroenterol. doi: 10.1097/MCG.0000000000001610 34516460

[B42] Surawicz C. M. Brandt L. J. Binion D. G. Ananthakrishnan A. N. Curry S. R. Gilligan P. H.. (2013). Guidelines for Diagnosis, Treatment, and Prevention of Clostridium Difficile Infections. Am. J. Gastroenterol. 108 (4), 478–498. doi: 10.1038/ajg.2013.4 23439232

[B43] Tang L. Feng W. Cheng J. Gong Y. (2020). Clinical Remission of Ulcerative Colitis After Different Modes of Faecal Microbiota Transplantation: A Meta-Analysis. Int. J. Colorectal Dis. 35 (6), 1025–1034. doi: 10.1007/s00384-020-03599-7 32388604

[B44] Vich V. A. Imhann F. Collij V. Jankipersadsing S. A. Gurry T. Mujagic Z.. (2018). Gut Microbiota Composition and Functional Changes in Inflammatory Bowel Disease and Irritable Bowel Syndrome. Sci. Transl. Med. 10 (472), eaap8914. doi: 10.1126/scitranslmed.aap8914 30567928

[B45] Witkowski M. Weeks T. L. Hazen S. L. (2020). Gut Microbiota and Cardiovascular Disease. Circ. Res. 127 (4), 553–570. doi: 10.1161/CIRCRESAHA.120.316242 32762536 PMC7416843

[B46] Yu E. W. Gao L. Stastka P. Cheney M. C. Mahabamunuge J. Torres Soto M.. (2020). Fecal Microbiota Transplantation for the Improvement of Metabolism in Obesity: The FMT-TRIM Double-Blind Placebo-Controlled Pilot Trial. PLos Med. 17 (3), e1003051. doi: 10.1371/journal.pmed.1003051 32150549 PMC7062239

[B47] Zhang F. Cui B. He X. Nie Y. Wu K. Fan D. (2018). Microbiota Transplantation: Concept, Methodology and Strategy for its Modernization. Protein Cell 9 (5), 462–473. doi: 10.1007/s13238-018-0541-8 29691757 PMC5960466

[B48] Zhou H. Y. Guo B. Lufumpa E. Li X. M. Chen L. H. Meng X.. (2021). Comparative of the Effectiveness and Safety of Biological Agents, Tofacitinib, and Fecal Microbiota Transplantation in Ulcerative Colitis: Systematic Review and Network Meta-Analysis. Immunol. Invest. 50 (4), 323–337. doi: 10.1080/08820139.2020.1714650 32009472

[B49] Zhu H. Mo Q. Shen H. Wang S. Liu B. Xu X. (2020). Carbohydrates, Glycemic Index, and Glycemic Load in Relation to Bladder Cancer Risk. Front. Oncol. 10, 530382. doi: 10.3389/fonc.2020.530382 33072566 PMC7538710

[B50] Zhu J. Yu X. Zheng Y. Li J. Wang Y. Lin Y.. (2020). Association of Glucose-Lowering Medications With Cardiovascular Outcomes: An Umbrella Review and Evidence Map. The Lancet. Diabetes Endocrinol. 8 (3), 192–205. doi: 10.1016/S2213-8587(19)30422-X 32006518

