# Supplementary material for: Fecal Microbiota Transplantation and Health Outcomes: An Umbrella Review of Meta-Analyses of Randomized Controlled Trials
Source: Front Cell Infect Microbiol. 2022 Jun 27;12:899845. doi: 10.3389/fcimb.2022.899845 (PMC9271871; doi:10.3389/fcimb.2022.899845)
Supplement: Supplementary file 1 [file DataSheet_1.pdf]

Supplemental material

Table 1. Detailed search query
Table 2. AMSTAR 2 evaluation method
Table 3. Excluded articles at full-text assessment (n=84)
Table 4. Associations of Fecal Microbiota Transplantation With Health Outcomes
Table 5. Summary of effects of fecal microbiota transplantation and health outcomes with detail of GRADE assessment

**Table 1. Detailed search query**
**Database:** PubMed<br>**Date limits: From inception to August 6, 2021**<br>Fecal Microbiota Transplantation[MeSH] Fecal Microbiota Transplantations [Title/Abstract] OR Microbiota Transplantation, Fecal [Title/Abstract] OR Microbiota Transplantations, Fecal [Title/Abstract] OR Transplantation, Fecal Microbiota [Title/Abstract] OR Transplantations, Fecal Microbiota [Title/Abstract] Intestinal Microbiota Transfer [Title/Abstract] OR Intestinal Microbiota Transfers [Title/Abstract] OR Microbiota Transfer, Intestinal [Title/Abstract] OR Microbiota Transfers, Intestinal [Title/Abstract] OR Transfer, Intestinal Microbiota [Title/Abstract] OR Transfers, Intestinal Microbiota [Title/Abstract] OR Fecal Transplantation [Title/Abstract] OR Fecal Transplantations [Title/Abstract] OR Transplantation, Fecal [Title/Abstract] OR Transplantations, Fecal [Title/Abstract] OR Fecal Transplant [Title/Abstract] OR Fecal Transplants [Title/Abstract] OR Transplant, Fecal [Title/Abstract] OR Transplants, Fecal [Title/Abstract] OR Donor Feces Infusion [Title/Abstract] OR Donor Feces Infusions [Title/Abstract] OR Feces Infusion, Donor [Title/Abstract] OR Feces Infusions, Donor [Title/Abstract] OR Infusion, Donor Feces [Title/Abstract] OR Infusions, Donor Feces [Title/Abstract] AND Systematic review[Title/Abstract]) OR (meta-analysis[Title/Abstract]
**Database:** Embase<br>**Date limits: From inception to August 6, 2021**<br>‘bacteriotherapy (feces)’:ab,ti OR ‘faecal bacteriotherapy’:ab,ti OR ‘faecal enema’:ab,ti OR ‘faecal infusion’:ab,ti OR ‘faecal matter transplant’:ab,ti OR ‘faecal microbial transplant’:ab,ti OR ‘faecal microbial transplantation’:ab,ti OR ‘faecal microbiome transplant’:ab,ti OR ‘faecal microbiome transplantation’:ab,ti OR ‘faecal microbiota transplant’:ab,ti OR ‘faecal microbiota transplantation’:ab,ti OR ‘faecal transplant’:ab,ti OR ‘faecal transplantation’:ab,ti OR ‘fecal bacterial transplant’:ab,ti OR ‘fecal bacterial transplantation’:ab,ti OR ‘fecal bacteriotherapy’:ab,ti OR ‘fecal enema’:ab,ti OR ‘fecal infusion’:ab,ti OR ‘fecal instillation’:ab,ti OR ‘fecal matter transplant’:ab,ti OR ‘fecal matter transplantation’:ab,ti OR ‘fecal microbe transplant’:ab,ti OR ‘fecal microbial transplant’:ab,ti OR ‘fecal microbial transplantation’:ab,ti OR ‘fecal microbiome transplant’:ab,ti OR ‘fecal microbiome transplantation’:ab,ti OR ‘fecal microbiota transplant’:ab,ti OR ‘fecal microbiotal transplant’:ab,ti OR ‘fecal microflora transplantation’:ab,ti OR ‘fecal transfusion’:ab,ti OR ‘fecal transplant’:ab,ti OR ‘fecal transplantation’:ab,ti OR ‘feces bacteriotherapy’:ab,ti OR ‘feces microbe transplantation’:ab,ti OR ‘feces microbiota transplantation’:ab,ti OR ‘feces microflora transplant’:ab,ti OR ‘feces microflora transplantation’:ab,ti OR ‘FMT (fecal microbiota transplantation)’:ab,ti OR ‘gut microbial transplant’:ab,ti OR ‘gut microbial transplantation’:ab,ti OR ‘gut microbiome transplant’:ab,ti OR ‘gut microbiome transplantation’:ab,ti OR ‘gut microbiota transplant’:ab,ti OR ‘gut microbiota transplantation’:ab,ti OR ‘gut microflora transplantation’:ab,ti OR ‘IMT (intestinal microbiota transplantation)’:ab,ti OR ‘intestinal microbe transplantation’:ab,ti OR ‘intestinal microbiota transplant’:ab,ti OR ‘intestinal microbiota transplantation’:ab,ti OR ‘intestinal microflora transplantation’:ab,ti OR ‘rectal bacteriotherapy’:ab,ti OR ‘stool enema’:ab,ti OR ‘stool infusion’:ab,ti OR ‘stool instillation’:ab,ti OR ‘stool microbial transplantation’:ab,ti OR ‘stool transplant’:ab,ti OR ‘stool transplantation’ AND ‘Systematic review’:ab,ti OR ‘meta-analysis’:ab,ti
**Database:** Cochrane<br>**Date limits: From inception to August 6, 2021**<br>‘bacteriotherapy (feces)’:ab,ti OR ‘faecal bacteriotherapy’:ab,ti OR ‘faecal enema’:ab,ti OR ‘faecal infusion’:ab,ti OR ‘faecal matter transplant’:ab,ti OR ‘faecal microbial transplant’:ab,ti OR ‘faecal microbial transplantation’:ab,ti OR ‘faecal microbiome transplant’:ab,ti OR ‘faecal microbiome transplantation’:ab,ti OR

‘faecal microbiota transplant’:ab,ti OR ‘faecal microbiota transplantation’:ab,ti OR ‘faecal transplant’:ab,ti OR ‘faecal transplantation’:ab,ti OR ‘fecal bacterial transplant’:ab,ti OR ‘fecal bacterial transplantation’:ab,ti OR ‘fecal bacteriotherapy’:ab,ti OR ‘fecal enema’:ab,ti OR ‘fecal infusion’:ab,ti OR ‘fecal instillation’:ab,ti OR ‘fecal matter transplant’:ab,ti OR ‘fecal matter transplantation’:ab,ti OR ‘fecal microbe transplant’:ab,ti OR ‘fecal microbial transplant’:ab,ti OR ‘fecal microbial transplantation’:ab,ti OR ‘fecal microbiome transplant’:ab,ti OR ‘fecal microbiome transplantation’:ab,ti OR ‘fecal microbiota transplant’:ab,ti OR ‘fecal microbiotal transplant’:ab,ti OR ‘fecal microflora transplantation’:ab,ti OR ‘fecal transfusion’:ab,ti OR ‘fecal transplant’:ab,ti OR ‘fecal transplantation’:ab,ti OR ‘feces bacteriotherapy’:ab,ti OR ‘feces microbe transplantation’:ab,ti OR ‘feces microbiota transplantation’:ab,ti OR ‘feces microflora transplant’:ab,ti OR ‘feces microflora transplantation’:ab,ti OR ‘FMT (fecal microbiota transplantation)’:ab,ti OR ‘gut microbial transplant’:ab,ti OR ‘gut microbial transplantation’:ab,ti OR ‘gut microbiome transplant’:ab,ti OR ‘gut microbiome transplantation’:ab,ti OR ‘gut microbiota transplant’:ab,ti OR ‘gut microbiota transplantation’:ab,ti OR ‘gut microflora transplantation’:ab,ti OR ‘IMT (intestinal microbiota transplantation)’:ab,ti OR ‘intestinal microbe transplantation’:ab,ti OR ‘intestinal microbiota transplant’:ab,ti OR ‘intestinal microbiota transplantation’:ab,ti OR ‘intestinal microflora transplantation’:ab,ti OR ‘rectal bacteriotherapy’:ab,ti OR ‘stool enema’:ab,ti OR ‘stool infusion’:ab,ti OR ‘stool instillation’:ab,ti OR ‘stool microbial transplantation’:ab,ti OR ‘stool transplant’:ab,ti OR ‘stool transplantation’ AND ‘Systematic review’:ab,ti OR ‘meta-analysis’:ab,ti

Table 2. AMSTAR 2 evaluation method.	
Grade	Standard
High	No or one non-critical weakness: the systematic review provides an accurate and comprehensive summary of the results of the available studies that address the question of interest.
Moderate	More than one non-critical weakness: the systematic review has more than one weakness but no critical flaws. It may provide an accurate summary of the results of the available studies that were included in the review.
Low	One critical flaw with or without non-critical weaknesses: the review has a critical flaw and may not provide an accurate and comprehensive summary of the available studies that address the question of interest.
Critically low	More than one critical flaw with or without non-critical weaknesses: the review has more than one critical flaw and should not be relied on to provide an accurate and comprehensive summary of the available studies.

Table 3. Excluded articles at full-text assessment (n=84)	
**First author**	**Reason of exclusion**
Madsen[1]	Non-meta-analysis
Tan[2]	Non-meta-analysis
Fehily[3]	Non-meta-analysis
Pession[4]	Non-meta-analysis
Leung[5]	Non-meta-analysis
Hammeken[6]	Non-meta-analysis
Shivaji[7]	Non-meta-analysis
Guilfoyle[8]	Non-meta-analysis
Marcella[9]	Non-meta-analysis
Martínez-González[10]	Non-meta-analysis
Chinna[11]	Non-meta-analysis
Yang[12]	Non-meta-analysis
Stalder[13]	Non-meta-analysis
Cold[14]	Non-meta-analysis
Hoilat[15]	Non-meta-analysis
Feuerstadt[16]	Non-meta-analysis
Kayal[17]	Non-meta-analysis
Zhang[18]	Non-meta-analysis
Du[19]	Non-meta-analysis
Pierrard[20]	Non-meta-analysis
Hammeken[21]	Non-meta-analysis
Saha[22]	Non-meta-analysis
Wright[23]	Non-meta-analysis
Iqbal[24]	Non-meta-analysis
Carrera-Quintanar[25]	Non-meta-analysis
Shogbesan[26]	Non-meta-analysis
Bafeta[27]	Non-meta-analysis
Wang[28]	Non-meta-analysis
Chapman[29]	Non-meta-analysis
Shogbesan[30]	Non-meta-analysis
Drekonja[31]	Non-meta-analysis
Rossen[32]	Non-meta-analysis
Wang[33]	Non-meta-analysis
Cammarota[34]	Non-meta-analysis
Drekonja[35]	Non-meta-analysis
Sha[36]	Non-meta-analysis
Guo[37]	Non-meta-analysis
Anderson[38]	Non-meta-analysis
**Recurrent Clostridioides difficile infection**	
Ramai[39]	Comparison of various methods of faecal microbiota transplantation
Tixier[40]	Only one randomized controlled trial
Du[41]	No control group (meta-analysis of proportions)
Tariq[42]	No outcome of interest
Pomares[43]	Comparison of various methods of faecal microbiota transplantation
Baunwall[44]	Not the maximum sample size
Tariq[45]	No control group (meta-analysis of proportions)
Tariq[46]	Abstract only
Hong[47]	Abstract only
Khan[48]	Comparison of various methods of Faecal microbiota transplantation
Tang[49]	Comparison of various methods of Faecal microbiota transplantation
Huq[50]	Abstract only
Quraishi[51]	Not the maximum sample size
Moayyedi[52]	Not the maximum sample size
Li[53]	No control group (meta-analysis of proportions)
Li[54]	No control group (meta-analysis of proportions)
Dakhoul[55]	No control group (meta-analysis of proportions)
Dodin[56]	Non-meta-analysis
Kassam[57]	No control group (meta-analysis of proportions)
Sofi[58]	No outcome of interest (follow-up after faecal microbiota transplantation)
Gough[59]	Case series
Sofi[60]	Case report
**Crohn's disease**	

Cheng[61]	No control group (meta-analysis of proportions)
**Antibiotic-resistant bacteria**	
Yoon[62]	No control group (meta-analysis of proportions)
Tavoukjian[63]	Case series
**Ulcerative colitis**	
Liu[64]	Not the maximum sample size
Dang[65]	Not the maximum sample size
Zhao[66]	Not the maximum sample size
Lam[67]	Not the maximum sample size
Feng[68]	Abstract only
Cao[69]	No control group (meta-analysis of proportions)
Narula[70]	Not the maximum sample size
Keshteli[71]	Letter
Costello[72]	Not the maximum sample size
Shi[73]	No control group (meta-analysis of proportions)
Sun[74]	No control group (meta-analysis of proportions)
Scaldaferri[75]	No outcome of interest
Green[76]	Not the maximum sample size
**Inflammatory bowel disease**	
Mocanu[77]	No control group (meta-analysis of proportions)
Imdad[78]	Not the maximum sample size
Chen[79]	No control group (meta-analysis of proportions)
Qazi[80]	No control group (meta-analysis of proportions)
Paramsothy[81]	Not the maximum sample size
Colman[82]	No control group (meta-analysis of proportions)
**Irritable Bowel Syndrome**	
Myneedu[83]	Not the maximum sample size
Xu[84]	Not the maximum sample size

Table 4. Associations of Fecal Microbiota Transplantation With Health Outcomes.											
**Source**	**Disease**	**Year**	**Comparison**	**No. of studies**	**Sample size**	**Duration of FMT (wks)**	**Outcome**	**Pooled effect estimates**	***P*-value**	**I^2(%)**	**GRADE rating**
Dharmaratne[85]	Antibiotic resistance burden	2021	FMT VS placebo	2	59	28	Clinical remission	RR = 4.90; 95% CI (1.92-12.50)	0.0003	0	Low
Fang[86]	Functional constipation	2021	FMT+ laxative vs laxative	2	163	4-12	Total effective rate	RR=1.33, 95% CI (1.10, 1.59)	0.003	13	Low
				3	206		BSFS score	MD=1.04, 95% CI (0.57, 1.51)	<0.0001	76	Very low
				2	146		Wexner score	MD=-3.25, 95% CI (-5.58, -0.92)	0.006	92	Very low
				2	160		KESS score	MD=-5.75, 95% CI (-7.64, -3.68)	<0.0001	0	Very low
				3	246		PAC-QOL score	MD=-18.56, 95% CI (-24.63, -10.68)	<0.0001	78	Very low
				NA	NA		Adverse effects	NA	NA	NA	
Caldeira[87]	Inflammatory bowel disease	2019	FMT vs placebo	6	355	7-12	Clinical remission	RR=1.70, 95% CI (1.12, 2.56)	0.029	45	Moderate
				6	355		Clinical response	RR=1.68, 95% CI (1.04, 2.72)	0.042	55	Moderate
				NA	NA		Adverse event	NA			
Tang[88]	Ulcerative colitis	2020	FMT vs placebo	7	431	4-48	Clinical remission	RR =1.50, 95% CI (1.06–2.12)	0.02	48	Moderate
				6	416		Adverse events	RR = 1.21, 95% CI (0.86–1.70)	0.27	0	Moderate
				4	267		Multi-donor	RR = 2.07, 95% CI (1.16–3.71)	0.01	41	Low
				2	76		Single-donor	RR = 1.30, 95% CI (0.90–1.73)	0.07	0	Low
				5	368		Lower digestive tract	RR = 1.68, 95% CI (1.09–2.59)	0.02	65	Low
				2	63		Up digestive tract	RR = 0.99, 95% CI (0.47–2.09)	0.97	0	Low
				4	263		Frozen feces	RR = 1.60, 95% CI (1.02-2.59)	0.04	59	Low
				2	90		Fresh feces	RR = 2.38, 95% CI	0.21	32	Low

								(0.62-9.11)			
Hui[89]	Clostridium difficile infection	2019	FMT vs placebo or vancomycin	8	537	8-24	Clinical remission	RR =1.82, 95% CI, 1.19–2.78	0.002	76	Moderate
							multiple infusions vs single infusion	RR =1.21, 95% CI, 1.08–1.37	0.001	0	Low
				NA	NA		Adverse events	NA			
Ianiro[90]	Irritable bowel syndrome	2019	FMT vs placebo	5	267	8-48	Clinical remission	RR= 0.98, 95% CI (0.58, 1.66)	0.94	78	Moderate
				3	159		Adverse events	RR=0.93, 95% CI (0.45, 1.92)	0.84	61	Moderate
				1	37		Adverse events (FMT via capsules)	RR=1.47, 95% CI (1.02, 2.12)	NA	NA	Very low
				2	107		Adverse events (FMT via colonoscopy)	RR=0.61, 95% CI (0.31, 1.22)	0.16	0	Very low
				2	100		FMT via oral capsules	RR=1.96, 95% CI (1.19-3.20)	0.008	14	Low
				2	103		FMT via colonoscopy	RR=0.63, 95% CI (0.43-0.93)	0.02	0	Low
				1	64		FMT via nasojejunal tube	RR=0.69, 95% CI (0.46-1.02)	0.06	NA	Very low
Proença[91]	Metabolic Syndrome	2020	FMT vs placebo	6	147	2-6	HbA1c	(MD = -1,32, 95% CI (-2.98, 0.34)	0.12	46	Moderate
				6	146		HDL cholesterol	(MD = 0,09, 95% CI (0.02, 0.16)	0.01	0	Moderate
				6	146		LDL cholesterol	(MD = 0.19, 95% CI (0,05, 0.34)	0.008	0	Moderate
				6	144		Fasting glucose	(MD = -0.09, 95% CI (-0,25, 0.07)	0.26	19	Moderate
				5	119		Triglycerides	(MD = -0.02, 95% CI (-0,52, 0.49)	0.95	91	Low
				5	124		Total cholesterol	(MD = 0.06, 95% CI (-0,10, 0.23)	0.47	0	Low
				3	78		BMI	(MD = -0.85, 95% CI (-2.90, 1.20)	0.42	70	Low
				4	100		Weight	(MD = 1.67, 95% CI (-9.50, 12.84)	0.77	86	Low

				4	105		HOMA-IR	(MD = -0.35, 95% CI (-1.56, 0.86)	0.57	69	Low
				NA	NA		Adverse events	NA	NA	NA	
				2	38	12	BMI	(MD = -0.34, 95% CI (-0.81, 1.13)	0.65	71	Low
				2	38		Hip width	(MD = -0.83, 95% CI (-4.68, 3.03)	0.67	0	Low
				3	65		Weight	(MD = 0.32, 95% CI (-6.81, 7.45)	0.93	0	Low
				2	43		HbA1c	(MD =-0.05, 95% CI (-2.17, 2.06)	0.96	0	Low
				2	43		LDL cholesterol	(MD =-0.32, 95% CI (-0.92, 0.29)	0.23	0	Low
				2	43		HDL cholesterol	(MD =0.49, 95% CI (-0.12, 1.10)	0.11	0	Low
				2	43		Triglycerides	(MD =0.07, 95% CI (-0.54, 0.57)	0.83	0	Low

Abbreviations: BSFS, bristol stool form scale; BMI, body mass index; FMT, fecal microbiota transplantation; HbA1c, Hemoglobin A1c; HDL, high density lipoprotein; HOMA-IR, homeostatic model assessment of insulin resistance; KESS, knowles eccersley scott symptom; LDL, low density lipoprotein; MD, mean difference; NA, not applicable; PAC-QOL, patient assessment of constipation quality of life uestionnaire; RR, risk ratio; 95%CI, 95% confidence intervals.

Table 5. Summary of effects of fecal microbiota transplantation and health outcomes with detail of GRADE assessment

Source	Disease	Outcome	No. of studies	Sample size	Duration of FMT (wks)	Metric	Random effect size (95% CI)	P value	GRADE evidence (Not serious (NS), serious (S), very serious (VS))					
									Risk of bias	Inconsistency, I^2, %	Indirectness	Imprecision	Publication bias	Overall certainty of evidence
Dharmaratne[85]	Antibiotic resistance burden	Clinical remission	2	59	28	RR	4.90; 95% CI (1.92-12.50)	0.0003	NS	NS 0	NS	NS	NA	Low
Fang [86]	Functional constipation	Total effective rate	2	163	4-12	RR	1.33, 95% CI (1.10, 1.59)	0.003	NS	NS 13	NS	NS	NA	Low
		BSFS score	3	206	4-12	MD	1.04, 95% CI (0.57, 1.51)	<0.0001	VS	VS 76	NS	NS	NA	Very low
		Wexner score	2	146	4-12	MD	-3.25, 95% CI (-5.58, -0.92)	0.006	VS	VS 92	NS	NS	NA	Very low
		KESS score	2	160	4-12	MD	-5.75, 95% CI (-7.64, -3.68)	<0.0001	VS	NS\ 0	NS	NS	NA	Very low
		PAC-QOL score	3	246	4-12	MD	-18.56, 95% CI (-24.63, -10.68)	<0.0001	VS	VS 78	NS	NS	NA	Very low
		Adverse effects	NA	NA	4-12		NA	NA		NA	NA	NA	NA	NA
Caldeira[87]	Inflammatory bowel disease	Clinical remission	6	355	7-12	RR	1.70, 95% CI (1.12, 2.56)	0.029	S	NS 45	NS	NS	NS 0.09	Moderate
		Clinical response	6	355	7-12	RR	1.68, 95% CI (1.04, 2.72)	0.042	S	S 55	NS	NS	NS 0.56	Moderate
		Adverse event	NA	NA	7-12		NA	NA	NA	NA	NA	NA		_NA
Tang [88]	Ulcerative colitis	Clinical remission	7	431	4-48	RR	1.50, 95% CI (1.06–2.12)	0.02	S	NS 48	NS	NS	NS 0.45	Moderate
		Adverse events	6	416	4-48	RR	1.21, 95% CI (0.86–1.70)	0.27	S	NS 0	NS	NS	NS 0.78	Moderate
		Multi-donor	4	267	4-48	RR	2.07, 95% CI (1.16–3.71)	0.01	VS	NS 41	NS	NS	S 0.02	Low

		Single-donor	2	76	4-48	RR	1.30, 95% CI (0.90–1.73)	0.07	VS	NS 0	NS	NS	NA	Low
		Lower digestive tract	5	368	4-48	RR	1.68, 95% CI (1.09–2.59)	0.02	VS	S 65	NS	NS	S 0.01	Low
		Up digestive tract	2	63	4-48	RR	0.99, 95% CI (0.47–2.09)	0.97	VS	NS 0	NS	NS	NA	Low
		Frozen feces	4	263	4-48	RR	1.60, 95% CI (1.02-2.59)	0.04	VS	S 59	NS	NS	NA	Low
		Fresh feces	2	90	4-48	RR	2.38, 95% CI (0.62-9.11)	0.21	VS	NS 32	NS	NS	NA	Low
Hui [89]	C. difficile infection	Clinical remission	8	537	8-24	RR	1.82, 95% CI, 1.19–2.78	0.002	S	VS 76	NS	NS	NS 0.89	Moderate
		multiple infusions vs single infusion	4	326	8-24	RR	1.21, 95% CI, 1.08–1.37	0.001	VS	NS 0	NS	NS	S 0.045	Low
		Adverse events	NA	NA	8-24		NA	NA	NA	NA	NA	NA	NA	NA
Ianiro [90]	Irritable bowel syndrome	Clinical remission	5	267	8-48	RR	0.98, 95% CI (0.58, 1.66)	0.94	S	VS\ 78	NS	NS	NS 0.89	Moderate
		Adverse events	3	159	8-48	RR	0.93, 95% CI (0.45, 1.92)	0.84	NS	S 61	NS	NS	NS 0.10	Moderate
		Adverse events (FMT via capsules)	1	37	8-48	RR	1.47, 95% CI (1.02, 2.12)	NA	VS	NA	VS	NS	NA	Very low
		Adverse events (FMT via colonoscopy)	2	107	8-48	RR	0.61, 95% CI (0.31, 1.22)	0.16	VS	NS 0	VS	NS	NA	Very low
		FMT via oral capsules	2	100	8-48	RR	1.96, 95% CI (1.19-3.20)	0.008	VS	NS 14	NS	NS	NA	Low
		FMT via colonoscopy	2	103	8-48	RR	0.63, 95% CI (0.43-0.93)	0.02	VS	NS 0	NS	NS	NA	Low
		FMT via nasojejunal tube	1	64	8-48	RR	0.69, 95% CI (0.46-1.02)	0.06	VS	NA	NS	NS	NA	Very low
Proença[91]	Metabolic Syndrome	HbA1c	6	147	2-6	MD	-1,32, 95% CI (-2.98, 0.34)	0.12	S	NS 46	NS	NS	NS 0.32	Moderate
		HDL cholesterol	6	146	2-6	MD	0,09, 95% CI (0.02, 0.16)	0.01	S	NS 0	NS	NS	NS 0.12	Moderate
		LDL cholesterol	6	146	2-6	MD	0.19, 95% CI (0,05, 0.34)	0.008	S	NS 0	NS	NS	NS 0.36	Moderate
		Fasting glucose	6	144	2-6	MD	-0.09, 95% CI (-0,25, 0.07)	0.26	S	NS 19	NS	NS	NS 0.06	Moderate

		Triglycerides	5	119	2-6	MD	-0.02, 95% CI (-0,52, 0.49)	0.95	VS	VS<br>91	NS	NS	NS<br>0.23	Low
		Total cholesterol	5	124	2-6	MD	0.06, 95% CI (-0,10, 0.23)	0.47	VS	NS<br>0	NS	NS	S<br>0.02	Low
		BMI	3	78	2-6	MD	-0.85, 95% CI (-2.90, 1.20)	0.42	VS	S<br>70	NS	NS	S<br>0.03	Low
		Weight	4	100	2-6	MD	1.67, 95% CI (-9.50, 12.84)	0.77	VS	VS<br>86	NS	NS	S<br>0.006	Low
		HOMA-IR	4	105	2-6	MD	-0.35, 95% CI (-1.56, 0.86)	0.57	VS	S<br>69	NS	NS	NS<br>0.28	Low
		Adverse events	NA	NA	2-6	MD	NA	NA	VS	NA	NS	NS		
		BMI	2	38	12	MD	-0.34, 95% CI (-0.81, 1.13)	0.65	VS	S<br>71	NS	NS	NA	Low
		Hip width	2	38	12	MD	-0.83, 95% CI (-4.68, 3.03)	0.67	VS	NS<br>0	NS	NS	NA	Low
		Weight	3	65	12	MD	0.32, 95% CI (-6.81, 7.45)	0.93		NS	NS	NS	NA	Low
		HbA1c	2	43	12	MD	-0.05, 95% CI (-2.17, 2.06)	0.96		0	NS	NS	NA	Low
		LDL cholesterol	2	43	12	MD	-0.32, 95% CI (-0.92, 0.29)	0.23		NS	NS	NS	NA	Low
		HDL cholesterol	2	43	12	MD	0.49, 95% CI (-0.12, 1.10)	0.11		0	NS	NS	NA	Low
		Triglycerides	2	43	12	MD	0.07, 95% CI (-0.54, 0.57)	0.83		NS<br>0	NS	NS	NA	Low

Abbreviations: BSFS, bristol stool form scale; BMI, body mass index; FMT, fecal microbiota transplantation; HbA1c, Hemoglobin A1c; HDL, high density lipoprotein; HOMA-IR, homeostatic model assessment of insulin resistance; KESS, knowles eccersley scott symptom; LDL, low density lipoprotein; MD, mean difference; NA, not applicable; PAC-QOL, patient assessment of constipation quality of life uestionnaire; RR, risk ratio; 95%CI, 95% confidence intervals.

References:

[1] Madsen M,Kimer N,Bendtsen F,Petersen AM. Fecal microbiota transplantation in hepatic encephalopathy: a systematic review.Scand J Gastroenterol.2021; 56,560-569.

[2] Tan Q,Orsso CE,Deehan EC,Kung JY,Tun HM,Wine E,Madsen KL,Zwaigenbaum L,Haqq AM. Probiotics, prebiotics, synbiotics, and fecal microbiota transplantation in the treatment of behavioral symptoms of autism spectrum disorder: A systematic review.Autism Res.2021.

[3] Fehily SR,Basnayake C,Wright EK,Kamm MA. Fecal microbiota transplantation therapy in Crohn's disease: Systematic review.J Gastroenterol Hepatol.2021.

[4] Pession A,Zama D,Muratore E,Leardini D,Gori D,Guaraldi F,Prete A,Turroni S,Brigidi P,Masetti R. Fecal Microbiota Transplantation in Allogeneic Hematopoietic Stem Cell Transplantation Recipients: A Systematic Review.J Pers Med.2021; 11.

[5] Leung J,Pham S. A Systematic Review of Fecal Microbiota Transplantation Versus Vancomycin for Treatment of Recurrent Clostridioides difficile Infection.Gastroenterol. Nurs.2021; 44,106-115.

[6] Hammeken LH,Baunwall S,Hvas CL,Ehlers LH. Health economic evaluations comparing faecal microbiota transplantation with antibiotics for treatment of recurrent Clostridioides difficile infection: a systematic review.Health Econ Rev.2021; 11,3.

[7] Shivaji S. A systematic review of gut microbiome and ocular inflammatory diseases: Are they associated?Indian J. Ophthalmol.2021; 69,535-542.

[8] Guilfoyle J,Considine J,Bouchoucha SL. Faecal microbiota transplantation and the patient experience: A systematic review.J. Clin. Nurs.2021; 30,1236-1252.

[9] Marcella C,Cui B,Kelly CR,Ianiro G,Cammarota G,Zhang F. Systematic review: the global incidence of faecal microbiota transplantation-related adverse events from 2000 to 2020.Alimentary Pharmacology and Therapeutics.2021; 53,33-42.

[10] Martínez-González AE,Andreo-Martínez P. Prebiotics, probiotics and fecal microbiota transplantation in autism: A systematic review.Rev Psiquiatr Salud Ment (Engl Ed).2020; 13,150-164.

[11] Chinna MA,Forth E,Wallace C,Milev R. Effect of fecal microbiota transplant on symptoms of psychiatric disorders: a systematic review.BMC Psychiatry.2020; 20,299.

[12] Yang J,Fu X,Liao X,Li Y. Effects of gut microbial-based treatments on gut microbiota, behavioral symptoms, and gastrointestinal symptoms in children with autism spectrum disorder: A systematic review.Psychiatry Res.2020; 293,113471.

[13] Stalder T,Kapel N,Diaz S,Grenouillet F,Koch S,Limat S,Daval F,Vuitton L,Nerich V. A systematic review of economic evaluation in fecal microbiota transplantation.Infect Control Hosp Epidemiol.2020; 41,458-466.

[14] Cold F,Kousgaard SJ,Halkjaer SI,Petersen AM,Nielsen HL,Thorlacius-Ussing O,Hansen LH. Fecal Microbiota Transplantation in the Treatment of Chronic Pouchitis: A Systematic Review.Microorganisms.2020; 8.

[15] Hoilat GJ,Durer C,Durer S,John S. Fecal microbiota transplant improves cognitive function in patients with hepatic encephalopathy: A systematic review of randomized clinical trial.Am. J. Gastroenterol.2020; 115,S519-S520.

[16] Feuerstadt P,Aroniadis OC,Svedlund FL,Garcia M,Ong K,Stong L,Boules M,Khanna S. HETEROGENEITY OF RANDOMIZED CONTROLLED TRIALS OF FECAL MICROBIOTA TRANSPLANTATION (FMT) IN RECURRENT CLOSTRIDIOIDES DIFFICILE INFECTION: A SYSTEMATIC REVIEW.Gastroenterology.2020; 158,990.

[17] Kayal M,Lambin T,Pinotti R,Dubinsky MC,Grinspan A. A systematic review of fecal microbiota transplant for the management of pouchitis.Crohn's and Colitis 360.2020; 2.
[18] Zhang Z,Mocanu V,Cai C,Dang J,Slater L,Deehan EC,Walter J,Madsen KL. Impact of Fecal Microbiota Transplantation on Obesity and Metabolic Syndrome-A Systematic Review.Nutrients.2019; 11.
[19] Du H,Kuang TT,Qiu S,Xu T,Gang HC,Fan G,Zhang Y. Fecal medicines used in traditional medical system of China: a systematic review of their names, original species, traditional uses, and modern investigations.Chin Med.2019; 14,31.
[20] Pierrard J,Seront E. Impact of the gut microbiome on immune checkpoint inhibitor efficacy-a systematic review.Curr. Oncol.2019; 26,395-403.
[21] Hammeken L,Jørgensen SMD,Dahlerup JF,Hvas CL,Ehlers LH. PGI33 THE COST-EFFECTIVENESS OF FAECAL MICROBIOTA TRANSPLANTATION VS. ANTIBIOTICS FOR PATIENTS WITH RECURRENT CLOSTRIDIOIDES DIFFICILE INFECTION: A SYSTEMATIC REVIEW.Value Health.2019; 22,S622.
[22] Saha S,Tariq R,Tosh PK,Pardi DS,Khanna S. Faecal microbiota transplantation for eradicating carriage of multidrug-resistant organisms: a systematic review.Clin. Microbiol. Infec.2019; 25,958-963.
[23] Wright ML,Fournier C,Houser MC,Tansey M,Glass J,Hertzberg VS. Potential Role of the Gut Microbiome in ALS: A Systematic Review.Biol. Res. Nurs.2018; 20,513-521.
[24] Iqbal U,Anwar H,Karim MA. Safety and efficacy of encapsulated fecal microbiota transplantation for recurrent Clostridium difficile infection: a systematic review.Eur J Gastroenterol Hepatol.2018; 30,730-734.
[25] Carrera-Quintanar L,Ortuño-Sahagún D,Franco-Arroyo NN,Viveros-Paredes JM,Zepeda-Morales AS,Lopez-Roa RI. The Human Microbiota and Obesity: A Literature Systematic Review of In Vivo Models and Technical Approaches.Int. J. Mol. Sci.2018; 19.
[26] Shogbesan O,Poudel DR,Victor S,Jehangir A,Fadahunsi O,Shogbesan G,Donato A. A Systematic Review of the Efficacy and Safety of Fecal Microbiota Transplant for Clostridium difficile Infection in Immunocompromised Patients.Can J Gastroenterol Hepatol.2018; 2018,1394379.
[27] Bafeta A,Yavchitz A,Riveros C,Batista R,Ravaud P. Methods and Reporting Studies Assessing Fecal Microbiota Transplantation: A Systematic Review.Ann. Intern. Med.2017; 167,34-39.
[28] Wang S,Xu M,Wang W,Cao X,Piao M,Khan S,Yan F,Cao H,Wang B. Systematic Review: Adverse Events of Fecal Microbiota Transplantation.PLoS One.2016; 11,e161174.
[29] Chapman BC,Moore HB,Overbey DM,Morton AP,Harnke B,Gerich ME,Vogel JD. Fecal microbiota transplant in patients with Clostridium difficile infection: A systematic review.J Trauma Acute Care Surg.2016; 81,756-764.
[30] Shogbesan O,Poudel D,Jehangir A,Fadahunsi O,Shogbesan G,Donato A. Fecal microbiota transplantation for clostridium difficile infections in immunocompromised patients: A systematic review.Am. J. Gastroenterol.2016; 111,S79.
[31] Drekonja D,Reich J,Gezahegn S,Greer N,Shaukat A,MacDonald R,Rutks I,Wilt TJ. Fecal Microbiota Transplantation for Clostridium difficile Infection: A Systematic Review.Ann. Intern. Med.2015; 162,630-638.
[32] Rossen NG,MacDonald JK,de Vries EM,D'Haens GR,de Vos WM,Zoetendal EG,Ponsioen CY. Fecal microbiota transplantation as novel therapy in gastroenterology: A systematic review.World J Gastroenterol.2015; 21,5359-5371.
[33] Wang SN,Xu MQ,Cao XC,Piao MY,Yan F,Cao HL,Wang BM. Systematic review: Adverse events of faecal microbiota transplantation.J. Digest. Dis.2015; 16,93.

[34] Cammarota G,Ianiro G,Gasbarrini A. Fecal microbiota transplantation for the treatment of Clostridium difficile infection: a systematic review.J. Clin. Gastroenterol.2014; 48,693-702.

[35] Drekonja D,Reich J,Gezahegn S,Greer N,Shaukat A,MacDonald R,Rutks I,Wilt T *Fecal Microbiota Transplantation for Clostridium Difficile Infection: A Systematic Review of the Evidence*; Department of Veterans Affairs (US): Washington (DC), 2014.

[36] Sha S,Liang J,Chen M,Xu B,Liang C,Wei N,Wu K. Systematic review: Faecal microbiota transplantation therapy for digestive and nondigestive disorders in adults and children.Alimentary Pharmacology and Therapeutics.2014; 39,1003-1032.

[37] Guo B,Harstall C,Louie T,Veldhuyzen VZS,Dieleman LA. Systematic review: faecal transplantation for the treatment of Clostridium difficile-associated disease.Aliment Pharmacol Ther.2012; 35,865-875.

[38] Anderson JL,Edney RJ,Whelan K. Systematic review: Faecal microbiota transplantation in the management of inflammatory bowel disease.Alimentary Pharmacology and Therapeutics.2012; 36,503-516.

[39] Ramai D,Zakhia K,Fields PJ,Ofosu A,Patel G,Shahnazarian V,Lai JK,Dhaliwal A,Reddy M,Chang S. Fecal Microbiota Transplantation (FMT) with Colonoscopy Is Superior to Enema and Nasogastric Tube While Comparable to Capsule for the Treatment of Recurrent Clostridioides difficile Infection: A Systematic Review and Meta-Analysis.Dig Dis Sci.2021; 66,369-380.

[40] Tixier EN,Verheyen E,Luo Y,Grinspan LT,Du CH,Ungaro RC,Walsh S,Grinspan AM. Systematic Review with Meta-Analysis: Fecal Microbiota Transplantation for Severe or Fulminant Clostridioides difficile.Dig Dis Sci.2021.

[41] Du C,Luo Y,Walsh S,Grinspan A. Oral Fecal Microbiota Transplant Capsules Are Safe and Effective for Recurrent Clostridioides difficile Infection: A Systematic Review and Meta-Analysis.J. Clin. Gastroenterol.2021; 55,300-308.

[42] Tariq R,Hayat M,Pardi D,Khanna S. Predictors of failure after fecal microbiota transplantation for recurrent Clostridioides difficile infection: a systematic review and meta-analysis.Eur J Clin Microbiol Infect Dis.2021; 40,1383-1392.

[43] Pomares BR,Veses V,Sheth CC. Effectiveness of fecal microbiota transplant for the treatment of Clostridioides difficile diarrhea: a systematic review and meta-analysis.Lett. Appl. Microbiol.2021; 73,149-158.

[44] Baunwall S,Lee MM,Eriksen MK,Mullish BH,Marchesi JR,Dahlerup JF,Hvas CL. Faecal microbiota transplantation for recurrent Clostridioides difficile infection: An updated systematic review and meta-analysis.EClinicalMedicine.2020; 29-30,100642.

[45] Tariq R,Pardi DS,Bartlett MG,Khanna S. Low Cure Rates in Controlled Trials of Fecal Microbiota Transplantation for Recurrent Clostridium difficile Infection: A Systematic Review and Meta-analysis.Clin. Infect. Dis.2019; 68,1351-1358.

[46] Tariq R,Furqan F,Jamshed S,Khanna S. Efficacy of Fecal Microbiota Transplant in Hematological Cancers Patients with Recurrent Clostridioides Difficile Infection: A Systemic Review and Meta-Analysis.Blood.2019; 134,5870.

[47] Hong AS,Yu WY,Hong JM,Azab M,Ohning GV,Jayaraj M. Proton pump inhibitor use on efficacy of fecal microbiota transplant administered by trans-oral routes for clostridioides difficile infection: A systematic review and analysis.Open Forum Infectious Diseases.2019; 6,S841-S842.

[48] Khan MY,Dirweesh A,Khurshid T,Siddiqui WJ. Comparing fecal microbiota transplantation to standard-of-care treatment for recurrent Clostridium difficile infection: a systematic review and meta-analysis.Eur J Gastroenterol Hepatol.2018; 30,1309-1317.

[49] Tang G,Yin W,Liu W. Is frozen fecal microbiota transplantation as effective as fresh fecal microbiota transplantation in patients with recurrent or refractory Clostridium difficile infection: A meta-analysis?Diagn Microbiol Infect Dis.2017; 88,322-329.

[50] Huq N,Kumaravel V,Affi A,Singh M. Factors influencing the success of FMT placed by colonoscopy: A systematic review and meta-analysis.Am. J. Gastroenterol.2017; 112,S97-S98.

[51] Quraishi MN,Widlak M,Bhala N,Moore D,Price M,Sharma N,Iqbal TH. Systematic review with meta-analysis: the efficacy of faecal microbiota transplantation for the treatment of recurrent and refractory Clostridium difficile infection.Alimentary Pharmacology and Therapeutics.2017; 46,479-493.

[52] Moayyedi P,Yuan Y,Baharith H,Ford AC. Faecal microbiota transplantation for Clostridium difficile-associated diarrhoea: A systematic review of randomised controlled trials.Med. J. Australia.2017; 207,166-172.

[53] Li YT,Ai LY,Wang ZH,Yan TT,Xu J,Fang JY. Long-term outcomes of fecal microbiota transplantation via different transplant routes for C. difficile infection.J. Digest. Dis.2016; 17,43.

[54] Li YT,Cai HF,Wang ZH,Xu J,Fang JY. Systematic review with meta-analysis: Long-term outcomes of faecal microbiota transplantation for Clostridium difficile infection.Alimentary Pharmacology and Therapeutics.2016; 43,445-457.

[55] Dakhoul L,Parikh K,Berkelhammer C. Fecal microbiota transplant in treatment of clostridium difficile colitis-pooled data analysis and a systematic review.Gastroenterology.2015; 148,S404.

[56] Dodin M,Katz DE. Faecal microbiota transplantation for Clostridium difficile infection.Int. J. Clin. Pract.2014; 68,363-368.

[57] Kassam Z,Lee CH,Yuan Y,Hunt RH. Fecal microbiota transplantation for Clostridium difficile infection: systematic review and meta-analysis.Am. J. Gastroenterol.2013; 108,500-508.

[58] Sofi AA,Silverman AL,Khuder S,Garborg K,Westerink JM,Nawras A. Relationship of symptom duration and fecal bacteriotherapy in Clostridium difficile infection-pooled data analysis and a systematic review.Scand J Gastroenterol.2013; 48,266-273.

[59] Gough E,Shaikh H,Manges AR. Systematic review of intestinal microbiota transplantation (fecal bacteriotherapy) for recurrent Clostridium difficile infection.Clin. Infect. Dis.2011; 53,994-1002.

[60] Sofi A,Nawras A,Sodeman T,Garborg K,Silverman A. Fecal bacteriotherapy works for clostridium difficile infection - A meta-analysis.Am. J. Gastroenterol.2011; 106,S161.

[61] Cheng F,Huang Z,Wei W,Li Z. Fecal microbiota transplantation for Crohn's disease: a systematic review and meta-analysis.Tech. Coloproctol.2021; 25,495-504.

[62] Yoon YK,Suh JW,Kang EJ,Kim JY. Efficacy and safety of fecal microbiota transplantation for decolonization of intestinal multidrug-resistant microorganism carriage: beyond Clostridioides difficile infection.Ann. Med.2019; 51,379-389.

[63] Tavoukjian V. Faecal microbiota transplantation for the decolonization of antibiotic-resistant bacteria in the gut: a systematic review and meta-analysis.J. Hosp. Infect.2019; 102,174-188.

[64] Liu X,Li Y,Wu K,Shi Y,Chen M. Fecal Microbiota Transplantation as Therapy for Treatment of Active Ulcerative Colitis: A Systematic Review and Meta-Analysis.Gastroenterol Res Pract.2021; 2021,6612970.

[65] Dang X,Xu M,Liu D,Zhou D,Yang W. Assessing the efficacy and safety of fecal microbiota transplantation and probiotic VSL#3 for active ulcerative colitis: A systematic review and meta-analysis.PLoS One.2020; 15,e228846.

[66] Zhao HL,Chen SZ,Xu HM,Zhou YL,He J,Huang HL,Xu J,Nie YQ. Efficacy and safety of fecal microbiota transplantation for treating patients with ulcerative colitis: A systematic review and meta-analysis.J Dig

Dis.2020; 21,534-548.

[67] Lam WC,Zhao C,Ma WJ,Yao L. The Clinical and Steroid-Free Remission of Fecal Microbiota Transplantation to Patients with Ulcerative Colitis: A Meta-Analysis.Gastroenterol Res Pract.2019; 2019,1287493.

[68] Feng X,Li S. Fecal microbial transplantation for the treatment of ulcerative colitis :a syetematic review and meta analysis.J. Gastroen. Hepatol.2019; 34,135.

[69] Cao Y,Zhang B,Wu Y,Wang Q,Wang J,Shen F. The Value of Fecal Microbiota Transplantation in the Treatment of Ulcerative Colitis Patients: A Systematic Review and Meta-Analysis.Gastroenterol Res Pract.2018; 2018,5480961.

[70] Narula N,Kassam Z,Yuan Y,Colombel JF,Ponsioen C,Reinisch W,Moayyedi P. Systematic Review and Meta-analysis: Fecal Microbiota Transplantation for Treatment of Active Ulcerative Colitis.Inflamm. Bowel Dis.2017; 23,1702-1709.

[71] Keshteli AH,Millan B,Madsen KL. Pretreatment with antibiotics may enhance the efficacy of fecal microbiota transplantation in ulcerative colitis: a meta-analysis.Mucosal Immunol.2017; 10,565-566.

[72] Costello SP,Soo W,Bryant RV,Jairath V,Hart AL,Andrews JM. Systematic review with meta-analysis: faecal microbiota transplantation for the induction of remission for active ulcerative colitis.Alimentary Pharmacology and Therapeutics.2017; 46,213-224.

[73] Shi Y,Dong Y,Huang W,Zhu D,Mao H,Su P. Fecal Microbiota Transplantation for Ulcerative Colitis: A Systematic Review and Meta-Analysis.PLoS One.2016; 11,e157259.

[74] Sun D,Li W,Li S,Cen Y,Xu Q,Li Y,Sun Y,Qi Y,Lin Y,Yang T,Xu P,Lu Q. Fecal Microbiota Transplantation as a Novel Therapy for Ulcerative Colitis: A Systematic Review and Meta-Analysis.Medicine (Baltimore).2016; 95,e3765.

[75] Scaldaferri F,Pecere S,Petito V,Zambrano D,Fiore L,Lopetuso LR,Schiavoni E,Bruno G,Gerardi V,Laterza L,Pizzoferrato M,Ianiro G,Stojanovic J,Poscia A,Papa A,Paroni SF,Sanguinetti M,Masucci L,Cammarota G,Gasbarrini A. Efficacy and Mechanisms of Action of Fecal Microbiota Transplantation in Ulcerative Colitis: Pitfalls and Promises From a First Meta-Analysis.Transplant Proc.2016; 48,402-407.

[76] Green JE,Davis JA,Berk M,Hair C,Loughman A,Castle D,Athan E,Nierenberg AA,Cryan JF,Jacka F,Marx W. Efficacy and safety of fecal microbiota transplantation for the treatment of diseases other than Clostridium difficile infection: a systematic review and meta-analysis.Gut Microbes.2020; 12,1-25.

[77] Mocanu V,Rajaruban S,Dang J,Kung JY,Deehan EC,Madsen KL. Repeated Fecal Microbial Transplantations and Antibiotic Pre-Treatment Are Linked to Improved Clinical Response and Remission in Inflammatory Bowel Disease: A Systematic Review and Pooled Proportion Meta-Analysis.J Clin Med.2021; 10.

[78] Imdad A,Nicholson MR,Tanner-Smith EE,Zackular JP,Gomez-Duarte OG,Beaulieu DB,Acra S. Fecal transplantation for treatment of inflammatory bowel disease.Cochrane Database Syst Rev.2018; 11,D12774.

[79] Chen T,Zhou Q,Zhang D,Jiang F,Wu J,Zhou JY,Zheng X,Chen YG. Effect of faecal microbiota transplantation for treatment of Clostridium difficile infection in patients with inflammatory bowel disease: A systematic review and meta-analysis of cohort studies.Journal of Crohn's and Colitis.2018; 12,710-717.

[80] Qazi T,Amaratunga T,Barnes EL,Fischer M,Kassam Z,Allegretti JR. The risk of inflammatory bowel disease flares after fecal microbiota transplantation: Systematic review and meta-analysis.Gut Microbes.2017; 8,574-588.

[81] Paramsothy S,Paramsothy R,Rubin DT,Kamm MA,Kaakoush NO,Mitchell HM,Castaño-Rodríguez N. Faecal microbiota transplantation for inflammatory bowel disease: A systematic review and

meta-analysis.Journal of Crohn's and Colitis.2017; 11,1180-1199.

[82] Colman RJ,Rubin DT. Fecal microbiota transplantation as therapy for inflammatory bowel disease: a systematic review and meta-analysis.J. Crohns Colitis.2014; 8,1569-1581.

[83] Myneedu K,Deoker A,Schmulson MJ,Bashashati M. Fecal microbiota transplantation in irritable bowel syndrome: A systematic review and meta-analysis.United European Gastroenterol J.2019; 7,1033-1041.

[84] Xu D,Chen VL,Steiner CA,Berinstein JA,Eswaran S,Waljee AK,Higgins P,Owyang C. Efficacy of Fecal Microbiota Transplantation in Irritable Bowel Syndrome: A Systematic Review and Meta-Analysis.Am. J. Gastroenterol.2019; 114,1043-1050.

[85] Dharmaratne P, Rahman N, Leung A, Ip M. Is there a role of faecal microbiota transplantation in reducing antibiotic resistance burden in gut? A systematic review and Meta-analysis. Annals of medicine (Helsinki). 2021; 53: 662-81.

[86] Fang S, Wu S, Ji L, Fan Y, Wang X, Yang K. The combined therapy of fecal microbiota transplantation and laxatives for functional constipation in adults. Medicine. 2021; 100: e25390.

[87] Caldeira LDF, Borba HH, Tonin FS, Wiens A, Fernandez-Llimos F, Pontarolo R. Fecal microbiota transplantation in inflammatory bowel disease patients: A systematic review and meta-analysis. Plos One. 2020; 15: e238910.

[88] Tang L, Feng W, Cheng J, Gong Y. Clinical remission of ulcerative colitis after different modes of faecal microbiota transplantation: A meta-analysis. Int J Colorectal Dis. 2020; 35: 1025-34.

[89] Hui W, Li T, Liu W, Zhou C, Gao F. Fecal microbiota transplantation for treatment of recurrent C. Difficile infection: An updated randomized controlled trial meta-analysis. Plos One. 2019; 14: e210016.

[90] Ianiro G, Eusebi LH, Black CJ, Gasbarrini A, Cammarota G, Ford AC. Systematic review with meta-analysis: Efficacy of faecal microbiota transplantation for the treatment of irritable bowel syndrome. Aliment Pharm Ther. 2019; 50: 240-8.

[91] Proença IM, Allegretti JR, Bernardo WM, de Moura DTH, Ponte Neto AM, Matsubayashi CO, et al. Fecal microbiota transplantation improves metabolic syndrome parameters: Systematic review with meta-analysis based on randomized clinical trials. Nutrition research.2020; 83: 1-14.
